# Synthesis of Tumor Selective Indole and 8-Hydroxyquinoline Skeleton Containing Di-, or Triarylmethanes with Improved Cytotoxic Activity

**DOI:** 10.3390/molecules29174176

**Published:** 2024-09-03

**Authors:** Dóra Hegedűs, Nikoletta Szemerédi, Krisztina Petrinca, Róbert Berkecz, Gabriella Spengler, István Szatmári

**Affiliations:** 1 Institute of Pharmaceutical Chemistry, University of Szeged, Eötvös u. 6, H-6720 Szeged, Hungary; 2 Department of Medical Microbiology, Albert Szent-Györgyi Health Center and Albert Szent-Györgyi Medical School, University of Szeged, Semmelweis utca 6, H-6725 Szeged, Hungaryspengler.gabriella@med.u-szeged.hu (G.S.); 3 Institute of Pharmaceutical Analysis, University of Szeged, Somogyi u. 4, H-6720 Szeged, Hungary; berkecz.robert@szte.hu; 4 Department of Forensic Medicine, Albert Szent-Györgyi Health Center, Kossuth Lajos sgt. 40, H-6724 Szeged, Hungary; 5 HUN-REN–SZTE Stereochemistry Research Group, University of Szeged, Eötvös u. 6, H-6720 Szeged, Hungary

**Keywords:** modified Mannich reaction, bioconjugation, 8-hydroxyquinoline skeleton, anticancer activity, indole skeleton

## Abstract

The reaction between glycine-type aminonaphthol derivatives substituted with 2- or 1-naphthol and indole or 7-azaindole has been tested. Starting from 2-naphthol as a precursor, the reaction led to the formation of ring-closed products, while in the case of a 1-naphthol-type precursor, the desired biaryl ester was isolated. The synthesis of a bifunctional precursor starting from 5-chloro-8-hydroxyquinoline, morpholine, and ethyl glyoxylate via modified Mannich reaction is reported. The formed Mannich base **10** was subjected to give bioconjugates with indole and 7-azaindole. The effect of the aldehyde component and the amine part of the Mannich base on the synthetic pathway was also investigated. In favor of having a preliminary overview of the structure-activity relationships, the derivatives have been tested on cancer and normal cell lines. In the case of bioconjugate **16**, as the most powerful scaffold in the series bearing indole and a 5-chloro-8-hydroxyquinoline skeleton, a potent toxic activity against the resistant Colo320 colon adenocarcinoma cell line was observed. Furthermore, this derivative was selective towards cancer cell lines showing no toxicity on non-tumor fibroblast cells.

## 1. Introduction

The Mannich reaction is a meaningful method in organic synthesis [1,2]. The modified Mannich reaction (*m*MR) is considered to be a particular variation of this C–C bond-forming reaction. 8-Hydroxyquinoline (8-HQ) as a unique electron-rich aromatic compound can be interpreted as a potential substrate of the modified Mannich reaction, and correlations between modification of the substitution pattern of the scaffold and biological activities were examined [3,4,5,6]. The indole skeleton proves to be a potent biological moiety through antitumor activity considering its effects on the proliferation, migration, invasion of different cancer cell lines [7], aryl hydrocarbon receptors [8], and ABCB1 efflux pump modifying [9] properties. It also exhibits immunoregulatory, anti-inflammatory, and antioxidative effects (lncRNAs and miRNAs expression) [10]. Additionally, symmetrical and un-symmetrical diindolylmethane derivatives were synthesized and tested at GPR84 (G protein-coupled receptor). It was found that the activity can be fine-tuned by substitution of indole skeleton [11].

The synthesis of indole- and naphthol-containing triarylmethane derivatives has been previously described. These compounds were synthesized by using different synthetic methods: (i) through the reaction of naphthol and the Mannich base of indole or (ii) applying the reaction of indole and the Mannich base of naphthol [12,13]. Recently, kynurenic acid derivatives were successfully applied as electron-rich aromatic compounds in this latter reaction [14].

As the result of our previous research, some C-3-coupled indole and azaindole derivatives showed toxicity on sensitive (Colo205) and ABCB1 efflux pump expressing (Colo320) colon adenocarcinoma cell lines and a normal, non-cancerous fibroblast cell line MRC-5 [15]. In addition, we previously studied the synthesis of new bifunctional precursors through the reaction of 2- or 1-naphthol, morpholine, and ethyl glyoxylate by using a modified Mannich-type synthetic method and their transformations via [4 + 2] cycloaddition with the use of different cyclic imines as dienophiles. Regarding the biological results, in the case of some compounds, inhibition of the efflux pump system in susceptible and methicillin-resistant *Staphylococcus aureus* strains was observed [16].

Based on the previous results, our first aim was the synthesis of a new glycine-type precursor bearing the 8-hydroxyquinoline skeleton. Our further aim was to investigate the reaction between some selected 8-hydroxyquinoline Mannich bases and indole or 7-azaindole forming new diarylmethane derivatives. Finally, their activity on Colo205 and Colo320 cell lines as well as on the normal MRC-5 cell line was also tested.

## 2. Results and Discussions

### 2.1. Synthesis

**Scheme 1:**
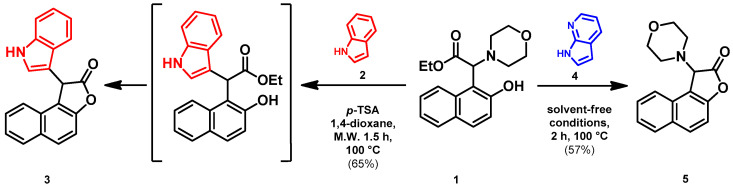
Reactions of 2-(2-hydroxynaphthalen-1-yl)-2-morpholinoacetate with indole and 7-azaindole.

Regarding the synthetic and biological results in the field of aminonaphthol and naphthoxazino derivatives [16] as well as compounds bearing the indole moiety [11,12,13,14,15], we focused our efforts on reacting ethyl 2-(2-hydroxynaphthalen-1-yl)-2-morpholinoacetate with indole and 7-azaindole (Scheme 1). Having in hand 2-naphthol-substituted glycine precursor **1** reported previously and indole (**2**), the reaction was performed at 100 °C in 1,4-dioxane under microwave conditions. *p*-Toluenesulfonic acid (*p*-TSA) is considered to be an acid catalyst, and is applied frequently in the modified Mannich reaction [17], therefore, we decided to investigate its effect on the reaction. After a reaction time of 1.5 h, the formation of **3** through an ester intermediate was observed. The synthesis of 1-(1*H*-indol-3-yl)naphtho[2,1-*b*]furan-2(1*H*)-one (**3**) was already described by Jadhav et al. utilizing a different synthetic pathway, starting from ethyl2-(4-fluorophenylamino)-2-(1*H*-indol-3-yl) acetate and 2-naphthol using scandium triflate in 1,2 dichloroethane via arylation-cyclization [18]. Applying different reaction conditions (modification of the solvent, temperature, and additive) led to the same product. In our next experiment, precursor **1** was reacted with 7-azaindole (**4**) in the presence of 10 mol% *p*-TSA at 100 °C under MW irradiation in 1,4-dioxane. Based on ^1^H NMR analysis of the crude reaction mixture and thin-layer chromatography (TLC), we concluded that the synthesis did not result in the desired derivative. The reaction was then repeated in toluene as the solvent, and the formation of a multi-spot reaction mixture was monitored by TLC. Unfortunately, the desired product could not be isolated even by using column chromatography. Next, the reaction was carried out in solvent-free conditions under microwave irradiation at 100 °C. A detailed NMR spectroscopic analysis of the product indicated the formation of a lactone structure (compound **5**) via intramolecular loss of ethanol. It is worth mentioning, that the formation and structure of this lactone as a side product was reported in our previous study [16].

**Scheme 2:**
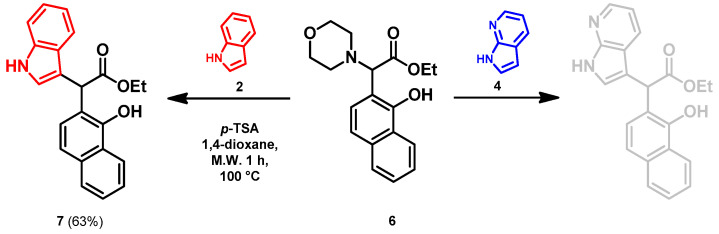
Preparation of target compound **7** from 1-naphthol-substituted precursor **6**.

In view of these preliminary findings, we focused on testing the reaction from 1-naphthol-substituted precursor **6** (Scheme 2). Accordingly, **6** and indole (**2**) were reacted in the presence of 10 mol% *p*-TSA at 100 °C for 60 min under MW irradiation in 1,4-dioxane. The progress of the synthesis was monitored by TLC showing the formation of a new spot. On the basis of ^1^H NMR analysis of the crude reaction mixture, the formation of **7** as a single product was assumed. On the basis of previous observations, the reaction was carried out under other conditions as well, applying toluene as the solvent in the presence of 10 mol% *p*-TSA. However, formation of the desired product did not take place. A similar result was found by running the reaction under solvent-free conditions. In favor of examining the possibility of extending the reaction scope, 7-azaindole (**4**) was reacted with precursor **6**. However, even by applying different solvents (toluene, 1,4-dioxane), using solvent-free conditions, and testing different temperatures (80 °C, 100 °C, 120 °C, 150 °C), the target compound did not form.

**Scheme 3:**
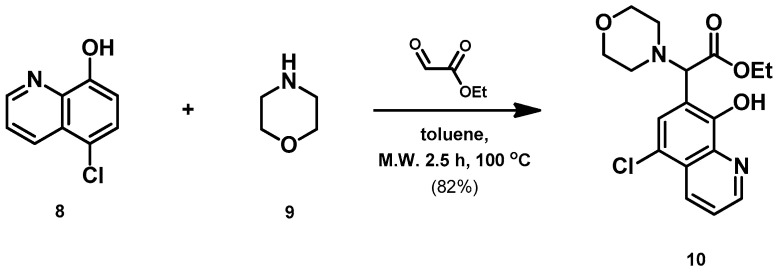
Synthesis of ethyl 2-(5-chloro-8-hydroxyquinolin-7-yl)-2-morpholinoacetate (**10**).

Based on literature data, 8-hydroxyquinoline is a biologically active moiety [5] interpreted as a potential 1-naphthol analogue. Chen et al. observed the most potent MMP-2/9 inhibitor effect, anti-proliferation activities against several cancer cell lines, and anti-migration/invasion activities on A549 cells in the case of Mannich bases of 5-chloro-8-hydroxyquinoline (5-Cl-8-HQ) [19]. Regarding earlier biological results, we proposed to examine the behavior of 5-Cl-8-HQ in the Mannich reaction. Accordingly, derivative **8** was reacted with morpholine (**9**) in the presence of ethyl glyoxylate as an aldehyde component (Scheme 3). According to TLC and NMR investigation of the crude reaction mixture, the formation of a single product and the presence of initial compounds were observed. New compound **10** was isolated and purified by crystallization and recrystallization from *i*Pr_2_O.

**Scheme 4:**
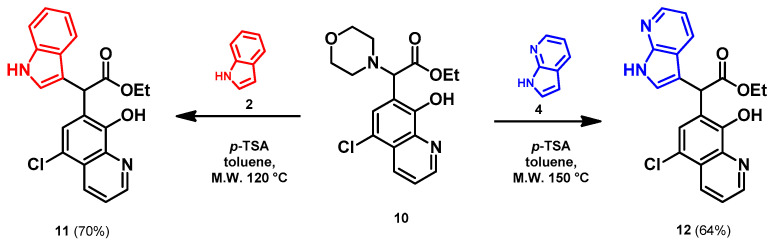
The transformation of compound **10** bearing the 5-chloro-8-hydroxyquinoline moiety with indole and 7-azaindole.

After introducing the newly synthesized precursor bearing the 8-hydroxyquinoline moiety, the preparation of **11** starting from **10** and indole (**2**) in the presence of 10 mol% *p*-TSA was accomplished (Scheme 4). In this case, **11** was isolated with a yield of 70% in 4 h at 120 °C. To study the scope and limitations of the reaction, the synthesis of **12** was planned through the reaction of the precursor **10** with 7-azaindole (**4**). The reaction was tested first in toluene at 120 °C by monitoring the conversion of the starting compounds by TLC analysis. Since the yield was not satisfactory, the reaction was repeated at 150 °C (Scheme 4). By using a higher temperature and a longer reaction time (3 h), the desired product was isolated by crystallization from the reaction mixture.

**Scheme 5:**
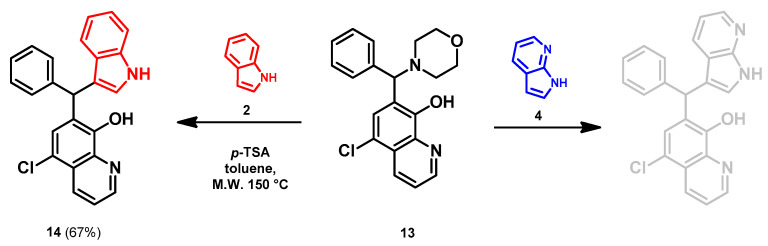
The reaction of precursor **13** with indole and 7-azaindole.

In order to study the effect of the aldehyde component on the synthetic pathway, 5-Cl-8-HQ and morpholine were fixed and the aldehyde moiety was varied among benzaldehyde and paraformaldehyde. The re-synthesis of precursor **13** was achieved based on the previously published synthetic method [20]. When precursor **13** was reacted with indole (**2**) at 150 °C for 4 h, the desired triarylmethane **14** was isolated in a yield of 67% after purification by column chromatography (Scheme 5). In contrast, the reaction between **13** and 7-azaindole (**4**) did not show any transformation upon testing various reaction conditions (solvent, reaction time, reaction temperature) (Scheme 5).

**Scheme 6:**
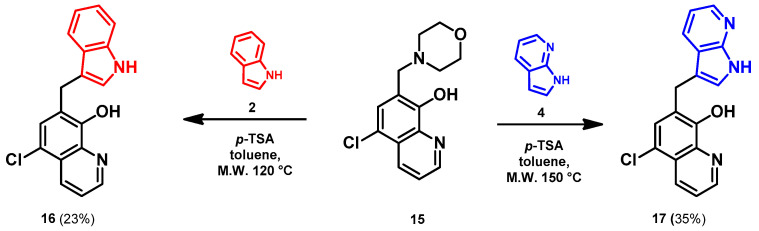
The transformation of **15** containing paraformaldehyde as the aldehyde component.

In the next step, we focused our efforts on investigating the transformations of precursors that were originally prepared from paraformaldehyde as the aldehyde component. For this reason, the re-synthesis of Mannich base **15** was accomplished by applying a synthetic method published previously [20]. Starting from precursor **15**, indole (**2**), and 7-azaindole (**4**), the formation of target compounds **16** and **17** were isolated in low yield with relatively long reaction times at high temperatures (Scheme 6).

**Scheme 7:**
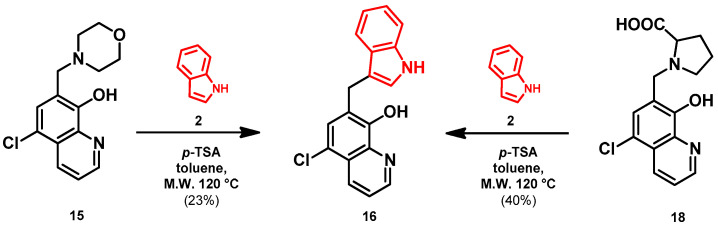
Synthesis of compound **16** starting from different precursors.

Since derivative **16** is postulated to be formed in the reaction of indole with *ortho*-quinone methide evolving from Mannich base **15**, we carried out a systematic investigation of the synthesis of **16**. According to previous observations, the amine moiety of the Mannich base can be interpreted as a leaving group. Consequently, two precursors have been selected. These were compound **15** bearing the morpholine skeleton and Mannich base **18** having the L-proline motif. This latter precursor was re-synthesized by using our synthetic pathway published earlier [21], starting from 5-chloro-8-hydroxyquinoline, L-proline, and aqueous formaldehyde. The parallel arylation of precursors **15** and **18** with indole was also investigated (Scheme 7).

**Figure 1:**
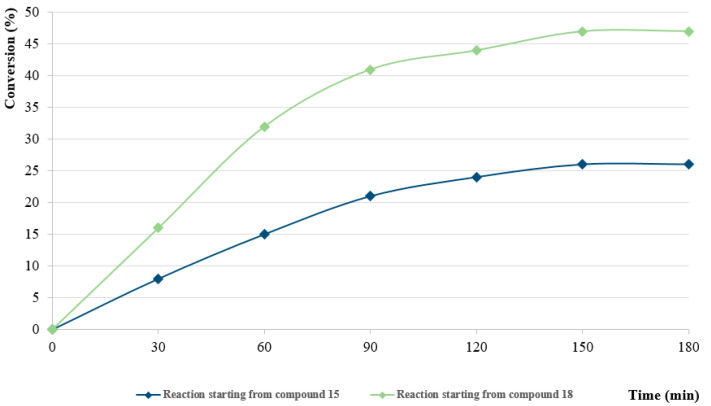
Systematic investigation of compounds **15** and **18** to determine the effect of the leaving groups.

This latter reaction offered a possibility to compare the effect of the leaving groups on conversion (Figure 1). The reaction was monitored by TLC and NMR analysis of the crude reaction mixtures every 30 min. These systematic investigations allowed us to conclude that starting from precursor **18** the desired diaryl derivative was formed in higher conversion. This may be explained by the better leaving group character of L-proline compared with that of morpholine.

### 2.2. Biological Evaluations

#### Cytotoxicity Assay

In this study, the potential ABCB1 cancer efflux pump-inhibiting activity of the de-rivatives was investigated. For this reason, the doxorubicin-sensitive Colo205 and the doxorubicin-resistant, ABCB1-expressing Colo320 colon adenocarcinoma cell lines were selected for biological studies. Based on the obtained results, the compounds showed a potent toxic activity against the sensitive Colo205 and resistant Colo320 cell lines (IC_50_ values were between 1.72 µM and 53.64 µM) (Table 1). Overall, it was observed that the tested compounds were more effective on the Colo320 cell line, with the exception of compound **14**. It is worth noting that **7** and **3** showed no effect on Colo205 cells, and **3** had no effect on Colo320 either. Only derivatives **11**, **12**, and **14** exerted a mild cytotoxic effect on the normal MRC-5 cell line compared to the cancer cell lines. This means that the compounds can be considered selective, as they were not toxic to normal fibroblast cells (Table 1). Doxorubicin was applied as a positive control, and DMSO was the solvent control. In our research, the selectivity of the compounds towards cancer cells (with the activity compared to normal cells) was investigated. The potential collateral sensitivity (CS) of the derivatives was also tested by applying sensitive Colo 205 colon adenocarcinoma cells and ABCB1- and LRP-expressing resistant Colo 320 cells [22]. The expression of the MDR transporter ABCB1 or P-glycoprotein is associated with a poor overall response to chemotherapy and unfavorable prognosis. Collateral sensitivity, which refers to the ability of certain compounds to selectively kill MDR cells over their parental counterparts, presents a potential alternative approach to overcoming MDR. This approach may lead to the development of highly selective and potent agents capable of effectively targeting MDR cells and resensitizing tumors to treatment [23]. 

Relative resistance (RR) values were calculated as the ratio between the IC_50_ of a compound against the resistant cells and the IC_50_ against the corresponding sensitive cells (Table 2). Compounds with RR < 1 show selectivity against resistant cells, whereas RR ≤ 0.5 means that the compounds are highly selective towards resistant cells. All derivatives, with the exception of **14**, were selective towards resistant tumor cells. Furthermore, **16** exerted high potency to eliminate the MDR Colo320 cells (RR = 0.37), and this derivative showed no toxicity on normal MRC-5 cells being the most powerful scaffold in the series.

Considering the correlation between structure and biological activity, it can be concluded in the case of powerful scaffolds that the biaryl structural element and 5-chloro-8-hydroxyquinoline skeleton could be identified as a significant moiety (compound **16**). For improved cytotoxic activity, the presence of a morpholine-bearing cationic centre and 5-chloro-8-hydroxyquinoline is beneficial (compound **10**). 

For comparison, in the case of C-3-indole- and azaindole-coupled cyclic amine derivatives, it was found that the simultaneous presence of the indole skeleton and thieno[3,2-*c*]pyridine or *β*-carboline is favourable for cytotoxic activity. The IC_50_ values were determined as 21.81 μM and 24.71 μM on the Colo205 cells, and 12.94 μM and 13.55 μM on the Colo320 cells [15]. Our present results support that the 5-chloro-8-hydroxyquinoline skeleton connected to the indole via methylene carbon is preferable in terms of cytotoxicity (Table 1, compound **16**; IC_50_: 13.06 μM on Colo205 cells and 4.87 μM on Colo320 cells) compared to the previously studied cyclic amines coupled to indole derivatives.

Furthermore, 8-hydroxyquinoline-D-proline and homo-proline hybrids and their half-sandwich Ru(η^6^-*p*-cymene) and Rh(η^5^-C_5_Me_5_) complexes were synthesized, and their in vitro cytotoxic activity on two human colon adenocarcinoma cell lines (Colo205 and Colo320) and on one non-tumoral human lung fibroblast cell line (MRC-5) were investigated by Pivarcsik et al. Regarding the biological activity, D-amino acid hybrids exhibited significant cytotoxicity (IC_50_ = 12–21 μM) against the tested cancer cell lines [24]. In parallel with our research, with Mannich base **10** having the morpholine instead of L-proline motif, it was observed that ethyl 2-(5-chloro-8-hydroxyquinolin-7-yl)-2-morpholinoacetate (**10**) showed a potent toxic activity against the doxorubicin-sensitive Colo205 (IC_50_ = 2.05 μM) and -resistant Colo320 (IC_50_ = 1.72 μM) cell lines, and no toxicity on normal MRC-5 cells. These results allowed us to conclude that morpholine as an amine component can improve the cytotoxic activity. Moreover, cytotoxic activity of substituted 2-(4-hydroxyquinolin-2-yl) acetates was evaluated using doxorubicin-sensitive and -resistant colon adenocarcinoma cell lines (Colo205 and Colo320, respectively) and normal human embryonic MRC-5 fibroblasts [25]. Interesting to note that for those compounds only the Knoevenagel products (para-substituted benzylidene derivatives) showed improved activity which supports that the presence of an 8-hydroxyquinoline skeleton is necessary for cytotoxic activity.

## 3. Materials and Methods

### 3.1. Biological Assays

#### 3.1.1. Cell Lines and Their Maintenance

The doxorubicin-sensitive Colo205 (ATCC-CCL-222) and the doxorubicin-resistant ABCB1- and LRP-expressing Colo320/MDR-LRP (ATCC-CCL-220.1) human colon adenocarcinoma cell lines were obtained from LGC Promochem in Teddington, UK. These cell lines were cultivated in RPMI 1640 medium supplemented with 10% heat-inactivated foetal bovine serum (FBS), 2 mM L-glutamine, 1 mM Na-pyruvate, 10 mM HEPES, along with nystatin, and gentamicin. The normal MRC-5 (ATCC CCL-171) human embryonic lung fibroblast cell line was purchased from Sigma-Aldrich (Merck KGaA, Darmstadt, Germany). The MRC-5 cells were cultured in EMEM medium, supplemented with 1% non-essential amino acid (NEAA) mixture, a selection of vitamins, 10% heat-inactivated FBS, 2 mM L-glutamine, 1 mM Na pyruvate, nystatin, and gentamicin. The cell lines were incubated in humidified atmosphere (5% CO_2_, 95% air) at 37 °C. The cells were detached with a Trypsin-Versene (EDTA) solution for 5 min at 37 °C.

#### 3.1.2. MTT Assay

The effects of increasing concentrations of the compounds on cell growth were tested in 96-well flat-bottomed microtiter plates. Namely, 1 × 10^4^ of human colonic adenocarcinoma cells in 100 μL of the medium (RPMI 1640) were added to each well, except for the medium control wells. The adherent human embryonic lung fibroblast cell line was seeded in the EMEM medium for 24 h before the assay. The two-fold serial dilutions of the compounds were made in a separate plate (100–0.19 μM) and then transferred to the plates containing the cells. The starting concentration of the solvent DMSO was 2% *v*/*v*, and the highest concentration of the compounds in the plate was 100 μM. Plates were incubated at 37 °C for 24 h. At the end of the incubation, 20 μL of MTT (thiazolyl blue tetrazolium bromide) solution (from a 5 mg/mL stock solution) was added to each well. After incubation at 37 °C for 4 h, 100 μL of sodium dodecyl sulfate (SDS) solution (10% SDS in 0.01 M HCl) was added to each well, and the plates were further incubated at 37 °C overnight. Cell growth was determined by measuring the optical density (OD) at 540 nm (ref. 630 nm) with a Multiscan EX ELISA reader (Thermo Labsystems, Cheshire, WA, USA). Inhibition of cell growth was expressed as IC_50_ values, defined as the inhibitory dose that reduces the growth of the cells exposed to the tested compounds by 50%. IC_50_ values and the SD of triplicate and duplicate (in the case of MRC-5) experiments were calculated by using GraphPad Prism software version 5.00 for Windows, with a non-linear regression curve fit (GraphPad Software, San Diego, CA, USA; www.graphpad.com, accessed on 12 July 2021). Doxorubicin (from a 2 mg/mL stock solution, Teva Pharmaceuticals) was used as a positive control. The solvent (DMSO) did not have any effect on the cell growth in the tested concentrations. The relative resistance (RR) was calculated as the ratio of the IC_50_ value in the resistant cancer cells and the IC_50_ value in the sensitive cancer cell lines. The selectivity indexes (SI) were calculated as the ratio of the IC_50_ value in the non-tumor cells and the IC_50_ value in the cancer cell lines. The activity of the compounds towards cancer cells is considered to be strongly selective if the selectivity index (SI) value is higher than 6, moderately selective if 3 < SI < 6, slightly selective if 1 < SI < 3, and non-selective if the SI is lower than 1 [22].

### 3.2. Preparation Protocols for the Synthesis of the New Derivatives

Melting points were determined using a Hinotek X-4 melting point apparatus. Merck Kieselgel 60F_254_ plates were applied for TLC (Merck KGaA, Darmstadt, Germany). Microwave reactions were carried out with a CEM Discover SP microwave reactor.

^1^H and ^13^C-NMR spectra were recorded in DMSO-d_6_ or CDCl_3_ solutions in 5 mm tubes at room temperature (RT) with a Bruker DRX-500 spectrometer (Bruker Biospin, Karlsruhe, Baden Wurttemberg, Germany) at 500 (^1^H) and 125 (^13^C) MHz, with the deuterium signal of the solvent as the lock and TMS as the internal standard (^1^H, ^13^C). All spectra (^1^H, ^13^C, and NOESY) were acquired and processed with the standard BRUKER software (TopSpin 3.6.2.).

The HRMS flow injection analysis was performed with Thermo Scientific™ Orbitrap Exploris™ 240 hybrid quadrupole-Orbitrap (Thermo Fisher Scientific, Waltham, MA, USA) mass spectrometer coupled to a Waters Acquity I-Class UPLC™ (Waters, Manchester, UK). The mass spectrometer was operated in negative (**7**) and positive (**10**, **11**, **12**, **14**, **16**, **17**) ionization mode.

The FTIR analysis was performed with Thermoscientific Nicolet Summit FTIR Spectrometer.

*Ethyl 2-(1-hydroxynaphthalen-2-yl)-2-(1H-indol-3-yl)acetate* (**7**)

In a 35 mL pressurized reaction vial, the mixture of ethyl 2-(1-hydroxynaphthalen-2-yl)-2-morpholinoacetate (**6**; 50.0 mg, 0.16 mmol) and indole (**2**; 17.5 mg, 0.16 mmol) in the presence of 10 mol% (2.8 mg, 0.016 mmol) *p*-TSA in 1,4-dioxane was heated at 100 °C for 1 h under MW irradiation. Following the removal of the solvent, the residue was purified by column chromatography (*n*-hexane:EtOAc, 4:1); R_f_ value = 0.32 (*n*-hexane:EtOAc, 4:1); 34.9 mg, (63%); oil; ^1^H-NMR (CDCl_3_): 1.35 (t, 3H, *J* = 7.15 Hz), 4.24–4.40 (m, 2H), 5.36 (s, 1H), 7.09 (t, 1H, *J* = 7.51 Hz), 7.19–7.21 (m, 1H), 7.34 (t, 2H, *J* = 8.86 Hz), 7.39 (d, 1H, *J* = 8.51 Hz), 7.44–7.52 (m, 3H), 7.74–7.80 (m, 1H), 8.08 (brs, 1H), 8.31–8.36 (m, 1H), 8.99 (s, 1H).^13^C NMR (CDCl_3_): 14.1; 29.7; 48.7; 111.1; 111.4; 115.9; 118.7; 119.9; 120.1; 122.5; 122.7; 123.6; 125.3; 126.3; 126.3; 126.5; 127.2; 128.9; 134.4; 136.5; 151.6; 176.0 (Figures S1 and S2). HRMS calculated for [M − H]^−^ = 344.1292 *m*/*z*, [2M − H]^−^ = 689.2657 *m*/*z* found 344.1287 *m*/*z* (Δm_i_ = −1.3 ppm) and 689.2659 *m*/*z* (Δm_i_ = 0.3 ppm). The ethoxy loss followed by ring-closing yielded proposed fragment ions with 298.0875 (Δmi = 0.3 ppm) *m*/*z* during in-source fragmentation (Figure S3). FTIR in Figure S4.

*Ethyl 2-(5-chloro-8-hydroxyquinolin-7-yl)-2-morpholinoacetate* (**10**)

A mixture of 5-chloro-8-hydroxyquinoline (**8**; 1.0 g, 5.56 mmol), ethyl glyoxylate (1.2 g, 5.93 mmol; 50% in toluene) and morpholine (**9**; 0.5 g, 5.54 mmol) in toluene (15 mL) was placed into a 35-mL pressurized reaction vial and heated at 100 °C for 2.5 h under MW irradiation. The solvent was removed in vacuo. The desired product was isolated by crystallization with EtOAc (10 mL), recrystallized from *i*Pr_2_O (5 mL); R_f_ value = 0.43 (CH_2_Cl_2_: EtOAc, 4:1); 1.6 g, (82%); white crystals; m.p. 124–126 °C; ^1^H-NMR (CDCl_3_): 1.23 (t, 3H, *J* = 7.12 Hz), 2.55–2.68 (m, 4H), 3.74–3.81 (m, 4H), 4.12–4.26 (m, 2H), 4.60 (s, 1H), 7.54–7.59 (m, 1H), 7.68 (s, 1H), 8.50 (d, 1H, *J* = 8.51 Hz), 8.87 (d, 1H, *J* = 4.36 Hz). ^13^C NMR (CDCl_3_): 14.1; 51.3; 61.4; 66.8; 68.2; 116.5; 120.8; 122.8; 126.3; 127.1; 133.2; 139.0; 149.0; 150.8; 170.1 (Figures S5 and S6). HRMS calculated for [M + H]^+^ =351.1106 *m*/*z*, [2M + Na]^+^ =723.1959 *m*/*z* found 351.1091 *m*/*z* (Δmi = −4.3 ppm) and 723.1927 *m*/*z* (Δmi = −4.4 ppm). The loss of the morpholino group can explain the observed 264.0411 *m*/*z* (Δmi = −4.2 ppm) peak during in-source fragmentation (Figure S7). FTIR in Figure S8.

*Ethyl 2-(5-chloro-8-hydroxyquinolin-7-yl)-2-(1H-indol-3-yl)acetate* (**11**)

Bifunctional precursor **10** (50.0 mg, 0.14 mmol) and indole (**2**; 17.0 mg, 0.14 mmol) in the presence of 10 mol% (2.4 mg, 0.014 mmol) *p*-TSA were dissolved in toluene (10 mL) in a 35 mL pressurized reaction vial and heated at 120 °C for 4 h under MW irradiation. Following column chromatography purification (EtOAc:*n*-hexane, 1:1), the eluent was removed in vacuo; R_f_ value = 0.63 (EtOAc:*n*-hexane, 1:1); 37.7 mg, (70%); oil; ^1^H-NMR (CDCl_3_): 1.27 (t, 3H, *J* = 7.16 Hz), 4.21–4.29 (m, 2H), 5.92 (s, 1H), 7.07 (t, 1H, *J* = 7.46 Hz), 7.19 (t, 1H, *J* = 7.65 Hz), 7.36–7.40 (m, 2H), 7.50–7.53 (m, 1H), 7.58–7.61 (m, 2H), 8.15 (brs, 1H), 8.45 (d, 1H, *J* = 8.49 Hz), 8.82 (d, 1H, *J* = 4.33 Hz). ^13^C NMR (CDCl_3_): 14.2; 41.4; 61.3; 111.2; 112.9; 119.1; 119.9; 120.4; 121.0; 122.3; 122.5; 122.9; 125.5; 126.7; 128.0; 133.3; 136.2; 138.5; 148.2; 148.4; 172.5 (Figures S9 and S10). HRMS calculated for [M + H]^+^ = 381.1000 *m*/*z*, [2M + Na]^+^ = 783.1748 *m*/*z* found 381.0986 *m*/*z* (Δmi = −3.7 ppm) and 783.1714 *m*/*z* (Δmi = −4.3 ppm) (Figure S11). FTIR in Figure S12.

*Ethyl 2-(5-chloro-8-hydroxyquinolin-7-yl)-2-(7-azaindole-3-yl)acetate* (**12**)

In a 35 mL pressurized reaction vial a mixture of product **10** (50.0 mg, 0.14 mmol) and 7-azaindole (**4**; 17.0 mg, 0.14 mmol) in the presence of 10 mol% (2.4 mg, 0.014 mmol) *p*-TSA in toluene was heated at 150 °C for 3 h under MW irradiation. The solvent was removed under reduced pressure. The desired product was isolated by crystallisation with Et_2_O (10 mL); R_f_ value = 0.52 (*n*-hexane:EtOAc, 1:4); 34.8 mg, (64%); beige crystals; m.p. 228–230 °C; ^1^H-NMR (DMSO): 1.16 (t, 3H, *J =* 7.11 Hz), 4.13–4.20 (q, 2H, *J =* 7.11 Hz), 5.73 (s, 1H), 7.03 (dd, 1H, *J_1_* = 4.64 Hz, *J_2_* = 7.91 Hz), 7.40 (s, 1H), 7.45–7.48 (m, 1H), 7.70–7.75 (m, 1H), 7.84 (d, 1H, *J =* 7.83 Hz), 8.22 (d, 1H, *J =* 4.79 Hz), 8.46 (d, 1H, *J =* 8.54 Hz), 8.98 (d, 1H, *J =* 4.25 Hz), 11.69 (brs, 1H). ^13^C NMR (DMSO): 14.5; 42.4; 61.3; 110.3; 115.9; 118.7; 119.0; 122.7; 123.5; 125.0; 125.3, 127.3; 127.9; 133.0, 139.1; 143.5; 149.0; 149.7; 150.2, 172.1 (Figures S13 and S14). HRMS calculated for [M + H]^+^ *m*/*z* = 382.0953, found *m*/*z* = 382.0939 (Δm_i_ = −3.7 ppm) (Figure S15). FTIR in Figure S16.

*7-((1H-indol-3-yl)(phenyl)methyl)-5-chloroquinolin-8-ol* (**14**)

Glycine type precursor **13** (50.0 mg, 0.14 mmol) and indole (**2**; 16.5 mg, 0.14 mmol) in the presence of 10 mol% (2.4 mg, 0.014 mmol) *p*-TSA in toluene were heated in a 35 mL pressurized reaction vial at 150 °C for 4 h under MW irradiation. Following the removal of the solvent, the residue was purified by column chromatography (*n*-hexane:EtOAc, 3:1); R_f_ value = 0.44 (*n*-hexane:EtOAc, 3:1); 36.2 mg, (67%); oil; ^1^H-NMR (CDCl_3_): 6.34 (s, 1H), 6.70 (s, 1H), 6.99 (t, 1H, *J =* 7.50 Hz), 7.17 (t, 1H, *J =* 7.33 Hz), 7.21–7.25 (m, 1H), 7.27–7.34 (m, 5H), 7.35–7.40 (m, 2H), 7.48–7.53 (m, 1H), 7.99 (brs, 1H), 8.46 (d, 1H, *J =* 8.42 Hz), 8.80 (d, 1H, *J =* 4.13 Hz). ^13^C NMR (CDCl_3_): 41.3; 111.1; 118.6; 119.5; 119.8; 120.0; 122.0; 122.3; 123.9; 125.0; 125.9; 126.4; 127.0; 128.4; 128.5; 128.9; 133.3; 136.8; 138.7; 142.8; 148.1; 148.4 (Figures S17 and S18). HRMS calculated for [M + H]^+^ *m*/*z* = 385.1102, found *m*/*z* = 385.1088 (Δm_i_= −3.6 ppm). The in-source fragmentation resulted in 268.0514 *m*/*z* (−3.7 ppm) of fragment ion, and the exit of indole moiety might be responsible for its formation (Figure S19). FTIR in Figure S20.

*General procedure for the synthesis of 7-((1H-indol-3-yl)methyl)-5-chloroquinolin-8-ol* (**16**) from precursor **15** and **18**

Indole (**2**; 20.0 mg, 0.17 mmol) and Mannich base **15** or **18** (0.17 mmol) in the presence of 10 mol% (2.9 mg, 0.017 mmol) *p*-TSA in toluene were heated at 120 °C for 3 h under MW irradiation. Following column chromatography purification (EtOAc:*n*-hexane, 1:2 in the case of reaction starting from precursor **15**; EtOAc:*n*-hexane, 1:3 in the case of reaction starting from precursor **18**), the eluent was removed in vacuo; R_f_ value = 0.68 (EtOAc:*n*-hexane, 1:2); 12.1 mg, (23%) in the case of reaction starting from precursor **15**; R_f_ value = 0.51 EtOAc:*n*-hexane, 1:3); 21.0 mg (40%) in the case of reaction starting from precursor **18**; oil; ^1^H-NMR (CDCl_3_): 4.32 (s, 2H), 7.06–7.11 (m, 2H), 7.18 (t, 1H, *J =* 7.44 Hz), 7.36 (d, 1H, *J =* 8.21 Hz), 7.43 (s, 1H), 7.46–7.51 (m, 1H), 7.65 (d, 1H, *J =* 7.88 Hz), 7.99 (brs, 1H), 8.45 (d, 1H, *J =* 8.39 Hz), 8.81 (d, 1H, *J =* 4.28 Hz). ^13^C NMR (CDCl_3_): 24.8; 111.1; 114.5; 119.2; 119.5; 120.0; 121.7; 122.2; 122.5; 123.4; 124.8; 127.4; 129.3; 133.3; 136.4; 138.6; 148.1; 148.3 (Figures S21 and S22). HRMS calculated for [M + H]^+^ 309.0789, found *m*/*z* = 309.0779 (Δm_i_ = −3.2 ppm) (Figure S23). FTIR in Figure S24.

*7-((7-azaindole-3-yl)methyl)-5-chloroquinolin-8-ol* (**17**)

A mixture of precursor **15** (50.0 mg, 0.18 mmol) and 7-azaindole (**4**; 21.0 mg, 0.18 mmol) in the presence of 10 mol% (3.1 mg, 0.018 mmol) *p*-TSA in toluene (15 mL) was placed into a 35-mL pressurized reaction vial and heated at 150 °C for 3 h under MW irradiation. Following column chromatography purification (EtOAc:*n*-hexane, 47:3), the eluent was removed in vacuo; oil; R_f_ value = 0.79 (EtOAc:*n*-hexane, 47:3); 19.5 mg, (35%); ^1^H-NMR (CDCl_3_): 5.68 (s, 2H), 6.48 (d, 1H, *J* = 3.33 Hz), 7.07–7.12 (m, 1H), 7.38 (d, 1H, *J* = 3.37 Hz), 7.45 (s, 1H), 7.49–7.54 (m, 1H), 7.93 (d, 1H, *J* = 7.71 Hz), 8.38 (d, 1H, *J* = 4.34 Hz), 8.45 (d, 1H, *J* = 8.20 Hz), 8.85 (s, 1H). ^13^C NMR (CDCl_3_): 42.5; 100.2; 115.9; 120.1; 120.7; 120.7; 122.5; 126.0; 128.1; 128.3; 129.2; 133.2; 139.2; 142.8; 147.4; 148.8; 149.4 (Figures S25 and S26). HRMS calculated for [M + H]^+^ *m*/*z* = 310.0742, found *m*/*z* = 310.0729 (Δm_i_ = −4.2 ppm) (Figure S27). FTIR in Figure S28.

## 4. Conclusions

Glycine derivatives bearing 2- and 1-naphthol were reacted with indole and 7-azaindole. Reactions were found to depend on the naphthol skeleton. Starting from the 2-naphthol-substituted precursor, lactam-type ring-closed products were isolated. The transformation of 1-naphthol, in turn, led to the formation of the desired biaryl ester **7**. Starting from 5-chloro-8-hydroxyquinoline, morpholine, and ethyl glyoxylate as the aldehyde component, a new bifunctional precursor was formed using a modified Mannich-type synthetic pathway. To further investigate the scope and limitations of the reaction, ethyl 2-(5-chloro-8-hydroxyquinolin-7-yl)-2-morpholinoacetate was reacted with indole and 7-azaindole resulting in the desired diarylmethanes **11** and **12**. The series of 8-hydroxyquinoline skeleton containing bioactive derivatives was extended by reacting 7-aminobenzyl-8-hydroxyquinoline and 7-aminomethyl-8-hydroxyquinoline Mannich bases with indole and 7-azaindole. The synthesis of 7-((1H-indol-3-yl)methyl)-5-chloroquinolin-8-ol from different precursors bearing the morpholine skeleton or L-proline as amines, was also achieved. These studies represent a systematic investigation of the effect of leaving groups on conversion. The reactions were performed either under microwave irradiation or solvent-free conditions. The effect of *p*-toluenesulfonic acid on the reactions was investigated. On the basis of biological evaluations, the synthesized compounds showed toxic activity against the Colo205 and Colo320 cell lines, being more toxic on the resistant cell line expressing the ABCB1 and LRP multidrug transporters. The possible interaction between the derivatives and the MDR transporters should be investigated by further functional and docking studies. Regarding the cytotoxic effect on the normal MRC-5 cell line, the compounds can be considered tumor-selective, exhibiting low or no toxicity on non-tumor fibroblasts. The reaction of 5-chloro-8-hydroxyquinoline, morpholine, and ethyl glyoxylate resulted in the potent toxic activity against the sensitive Colo205 and resistant Colo320 cell lines. The compound can be considered selective, as it was not toxic to normal fibroblast cells. Besides the presence of 5-chloro-8-hydroxyquinoline, the morpholine moiety furnishing a cationic centre have been found to be relevant in anticancer activity. 7-((1H-indol-3-yl)methyl)-5-chloroquinolin-8-ol was detected as the most powerful scaffold in the series. This derivative exerted high potency to eliminate the resistant Colo320 cells and showed no toxicity on normal MRC-5 cells. The biaryl structure bearing 5-chloro-8-hydroxyquinoline and an indole skeleton can be considered favorable in the aspect of toxic activity against cancer cell lines.

## Figures and Tables

**Table 1:** Cytotoxic effect of the compounds on sensitive (Colo205) and resistant (Colo320) colon adenocarcinoma, and MRC-5 normal embryonal fibroblast cell lines based on the IC_50_ values. Doxorubicin was used as a positive control. AV: average of 3 or 2 parallel experiments, SD: standard deviation. DMSO was applied as a solvent control that had no toxic effect at the highest concentration ap-plied in the assay (2% (*v*/*v*) concentration).

	Colo205 (µM)					Colo320 (µM)				MRC-5 (µM)		
					AV ± SD				AV ± SD			AV ± SD
**3**	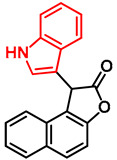	-	-	-	>100 ± -	-	-	-	>100 ± -	-	-	>100 ± -
**7**	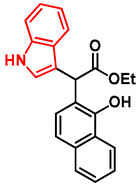	-	-	-	>100 ± -	53.82	54.92	52.18	53.64 ± 1.38	-	-	>100 ± -
**10**	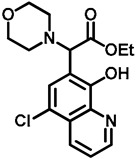	2.07	2.06	2.02	2.05 ± 0.02	1.565	1.813	1.79	1.72 ± 0.14	-	-	>100 ± -
**11**	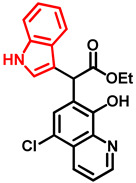	5.51	4.71	4.42	4.88 ± 0.56	4.636	4.832	4.957	4.81 ± 0.16	54.46	55.08	54.77 ± 0.44
**12**	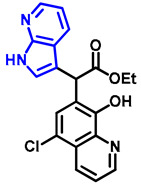	8.05	7.42	8.84	8.11 ± 0.71	5.157	4.923	5.109	5.06 ± 0.12	46.9	45.56	46.23 ± 0.95
**14**	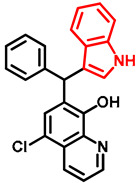	2.49	2.49	2.63	2.53 ± 0.08	2.552	2.84	2.781	2.72 ± 0.15	30.08	29.28	29.68 ± 0.57
**16**	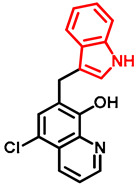	13.56	13.37	12.26	13.06 ± 0.70	4.967	4.74	4.91	4.87 ± 0.12	-	-	>100 ± -
**17**	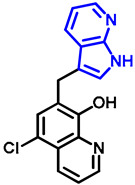	36.49	39.42	40.40	38.77 ± 2.03	22.98	21.89	22.54	22.47 ± 0.55	-	-	>100 ± -
	**DOX**	0.84	0.89	0.81	0.85 ± 0.04	3.117	3.12	2.961	3.07 ± 0.09	-	-	>8.62 ± -
	**DMSO**	-	-	-	>2% ± -	-	-	-	>2% ± -	-	-	>2% ± -

**Table 2:** Selectivity indexes (SI) and relative resistance (RR) of the compounds. NA: not applicable (if the precise value cannot be determined because of the lack of a precise IC_50_).

		MRC-5/Colo205	MRC-5/Colo320	Colo320/Colo205
		SI		RR
**3**	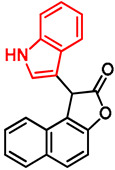	-	-	NA
**7**	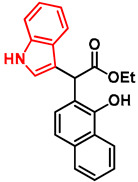	-	NA	NA
**10**	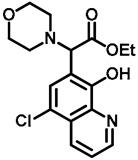	>6	>6	0.83
**11**	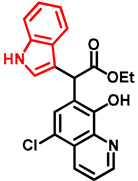	11.2	11.4	0.98
**12**	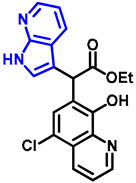	5.7	9.1	0.62
**14**	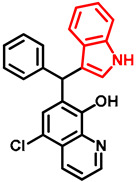	11.7	11	1.07
**16**	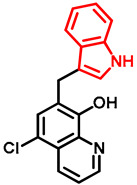	>6	>6	0.37
**17**	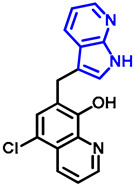	NA	NA	0.57

## Data Availability

The study did not report data.

## Supplementary Materials

The ^1^H, ^13^C NMR spectra, FTIR spectra and HRMS spectra as supporting information can be downloaded at: https://www.mdpi.com/article/10.3390/molecules29174176/s1.
