# Supplementary material for: Synthesis of Tumor Selective Indole and 8-Hydroxyquinoline Skeleton Containing Di-, or Triarylmethanes with Improved Cytotoxic Activity
Source: Molecules. 2024 Sep 3;29(17):4176. doi: 10.3390/molecules29174176 (PMC11396803; doi:10.3390/molecules29174176)
Supplement: Supplementary file 1 [file molecules-29-04176-s001.zip › molecules-3169900-supplementary.pdf]

Synthesis of tumor selective indole and 8-hydroxyquinoline skeleton containing di-, or triarylmethanes with improved cytotoxic activity

Dóra Hegedűs [1], Nikoletta Szemerédi [2], Krisztina Petrinca [1], Róbert Berkecz [3,4], Gabriella Spengler [2] and István Szatmári [1,5]*

[1] Institute of Pharmaceutical Chemistry, University of Szeged, H-6720 Szeged, Eötvös u. 6, Hungary; szatmari.istvan@szte.hu
[2] Department of Medical Microbiology, Albert Szent-Györgyi Health Center and Albert Szent-Györgyi Medical School, University of Szeged, Semmelweis utca 6, 6725 Szeged, Hungary; spengler.gabriella@med.u-szeged.hu
[3] Institute of Pharmaceutical Analysis, University of Szeged, H-6720 Szeged, Somogyi u. 4, Hungary, berkecz.robert@szte.hu
[4] Department of Forensic Medicine, Albert Szent-Györgyi Health Centre, H-6724 Szeged, Kossuth Lajos sgt. 40, Hungary, berkecz.robert@szte.hu
[5] HUN-REN–SZTE Stereochemistry Research Group, University of Szeged, H-6720 Szeged, Eötvös u. 6, Hungary
* Correspondence: szatmari.istvan@szte.hu

Contents

1. ^{1}H NMR, ^{13}C NMR, HRMS, FTIR spectra of synthesized compounds........................2.

Ethyl 2-(1-hydroxynaphthalen-2-yl)-2-(1*H*-indol-3-yl)acetate (7)

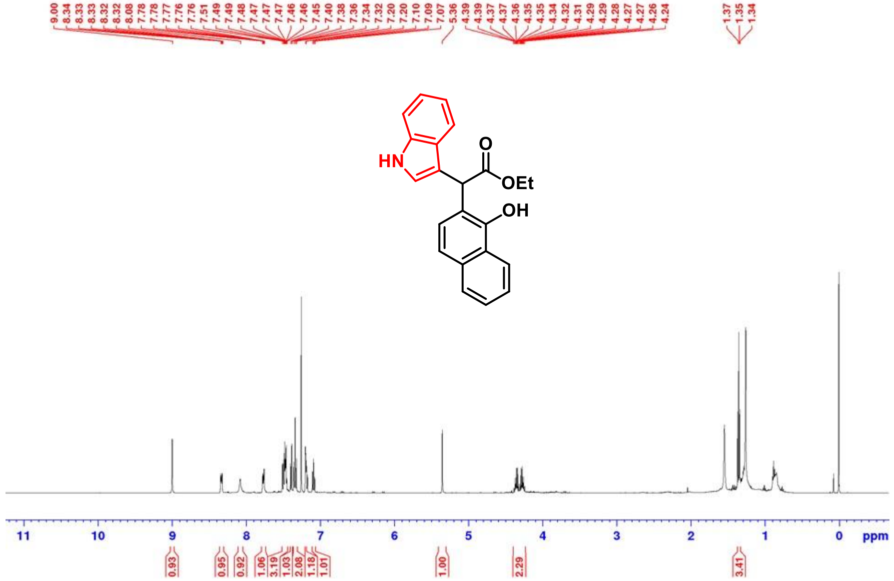

Figure S1. ^{1}H-NMR spectrum of **7**.

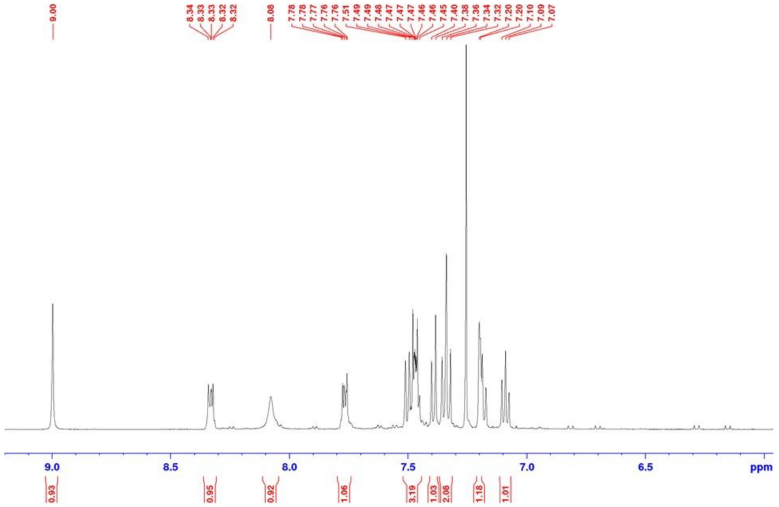

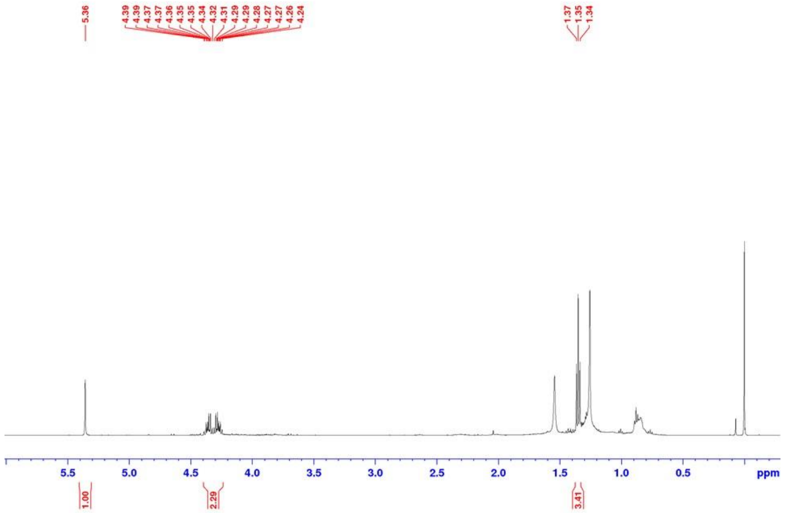

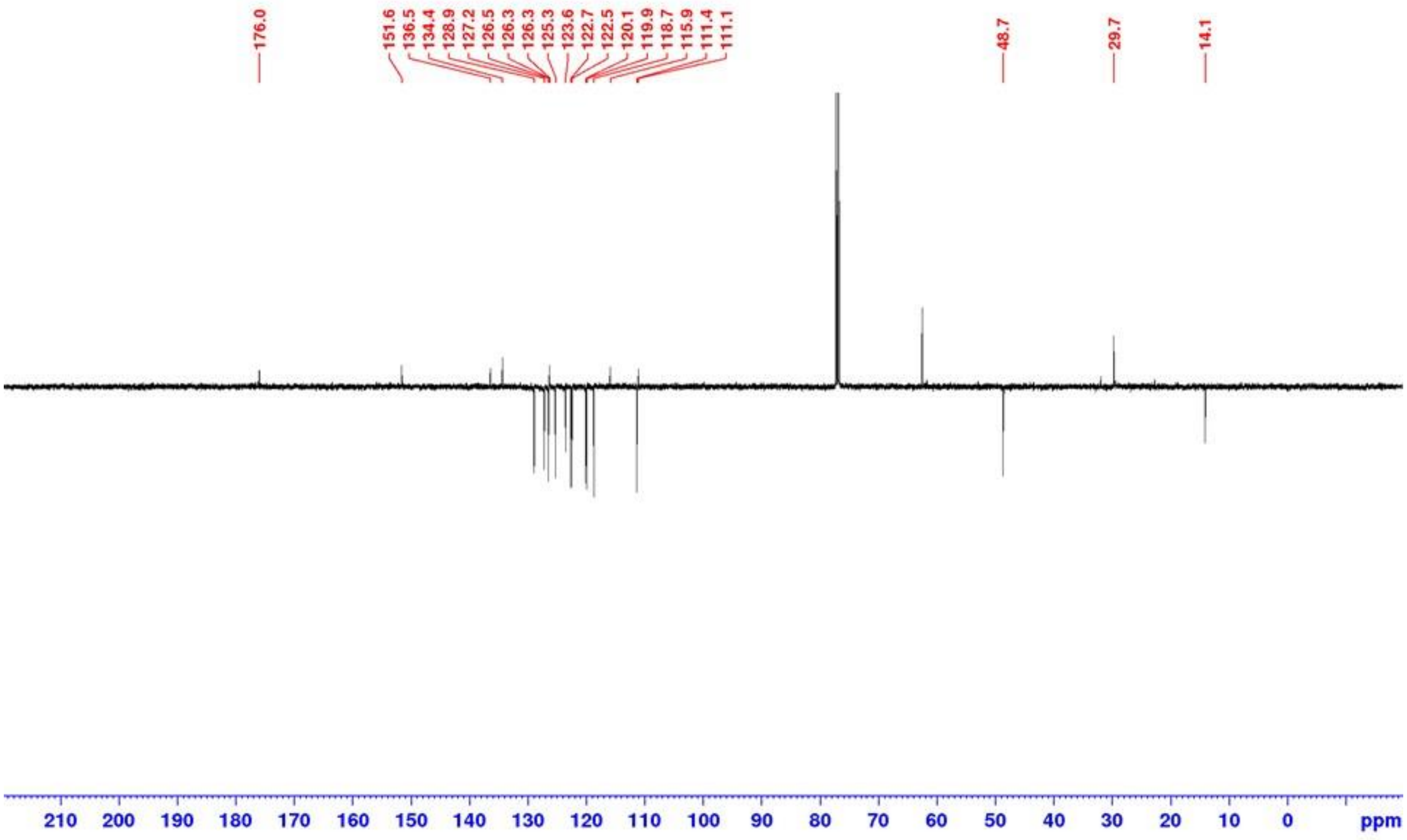

Figure S2. ^{13}C-NMR spectrum of **7**.

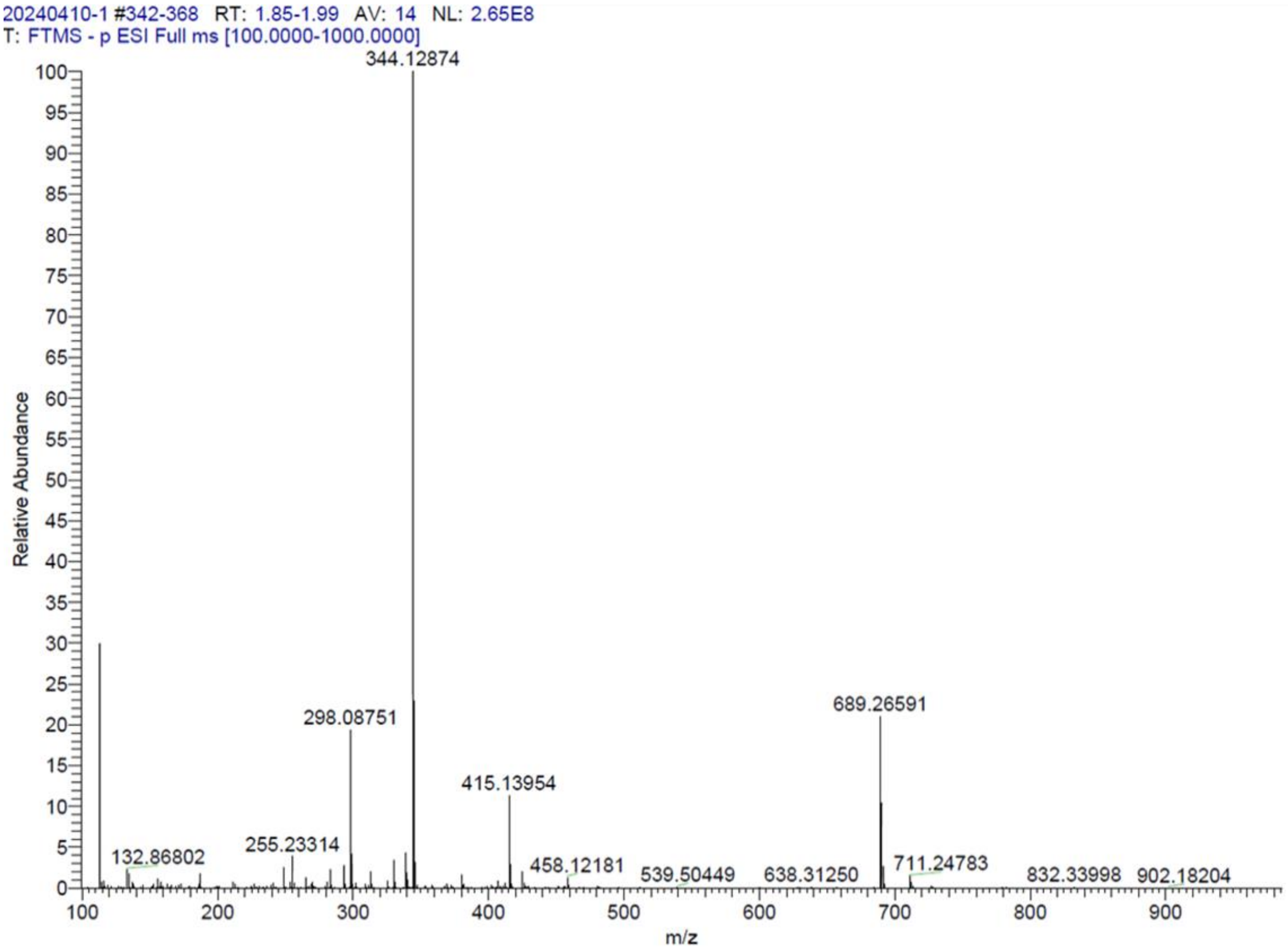

Figure S3. HRMS spectrum of **7** in negative mode.

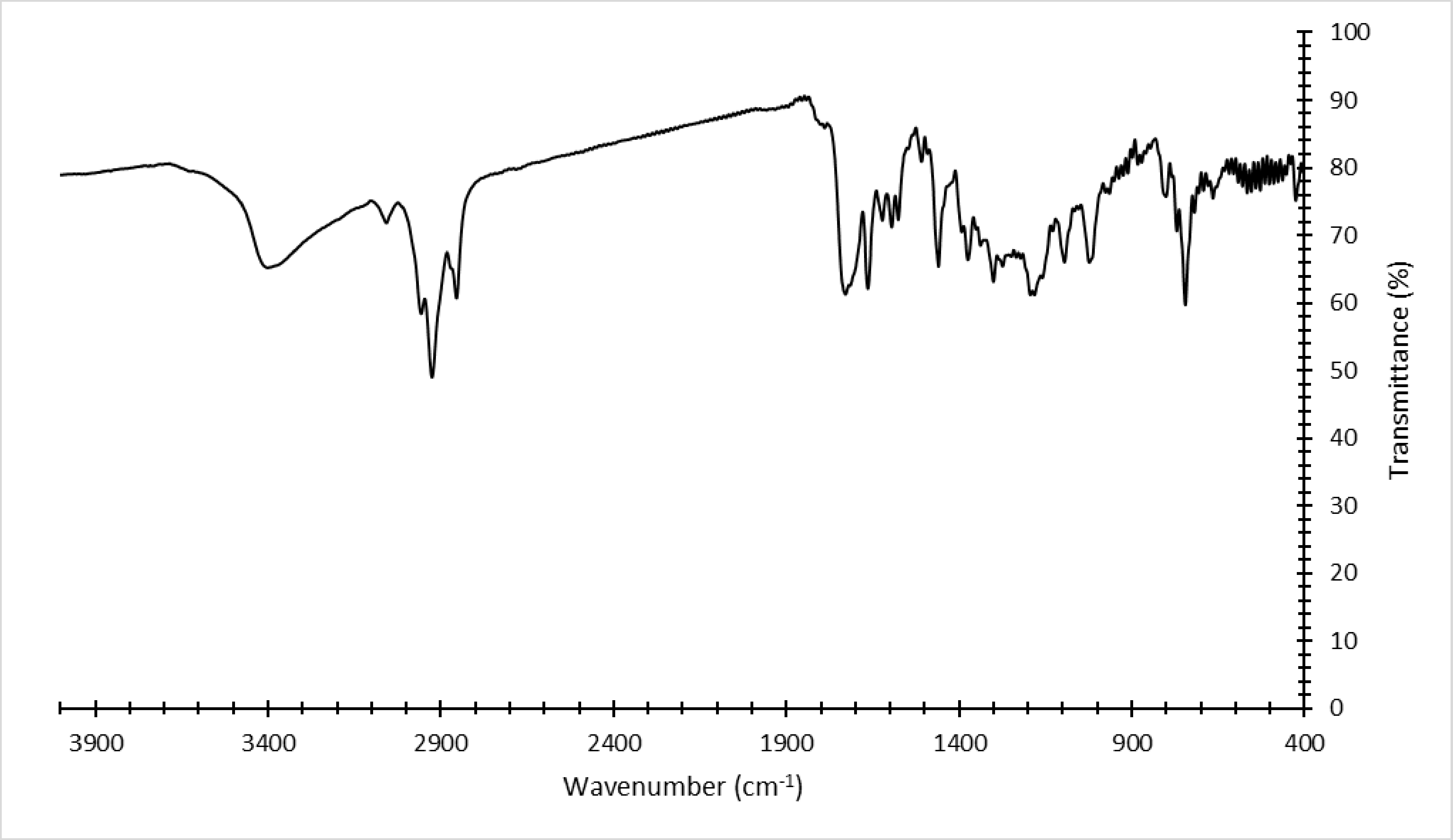

Figure S4. FTIR spectrum of **7**.

Ethyl 2-(5-chloro-8-hydroxyquinolin-7-yl)-2-morpholinoacetate (10)

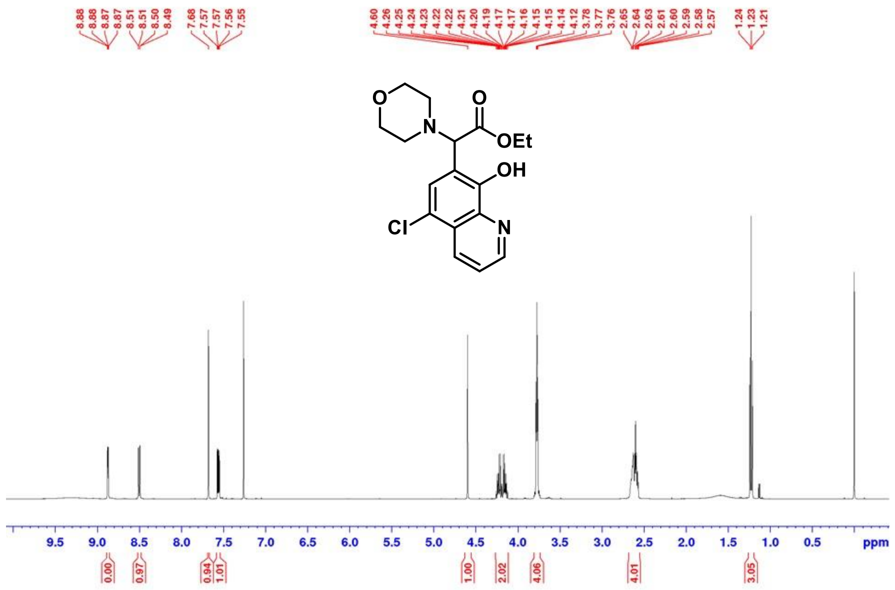

Figure S5. ^{1}H-NMR spectrum of **10**.

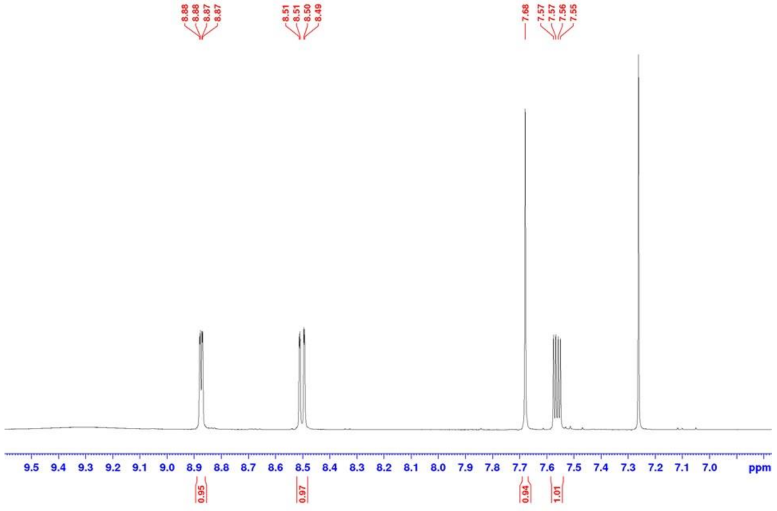

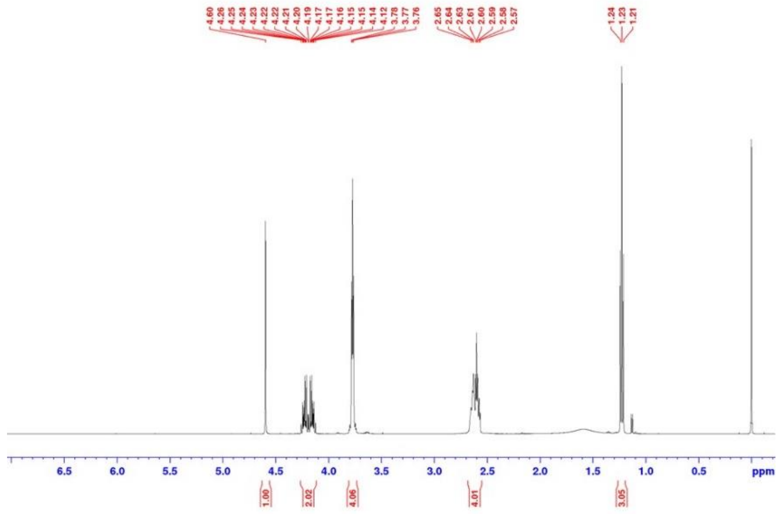

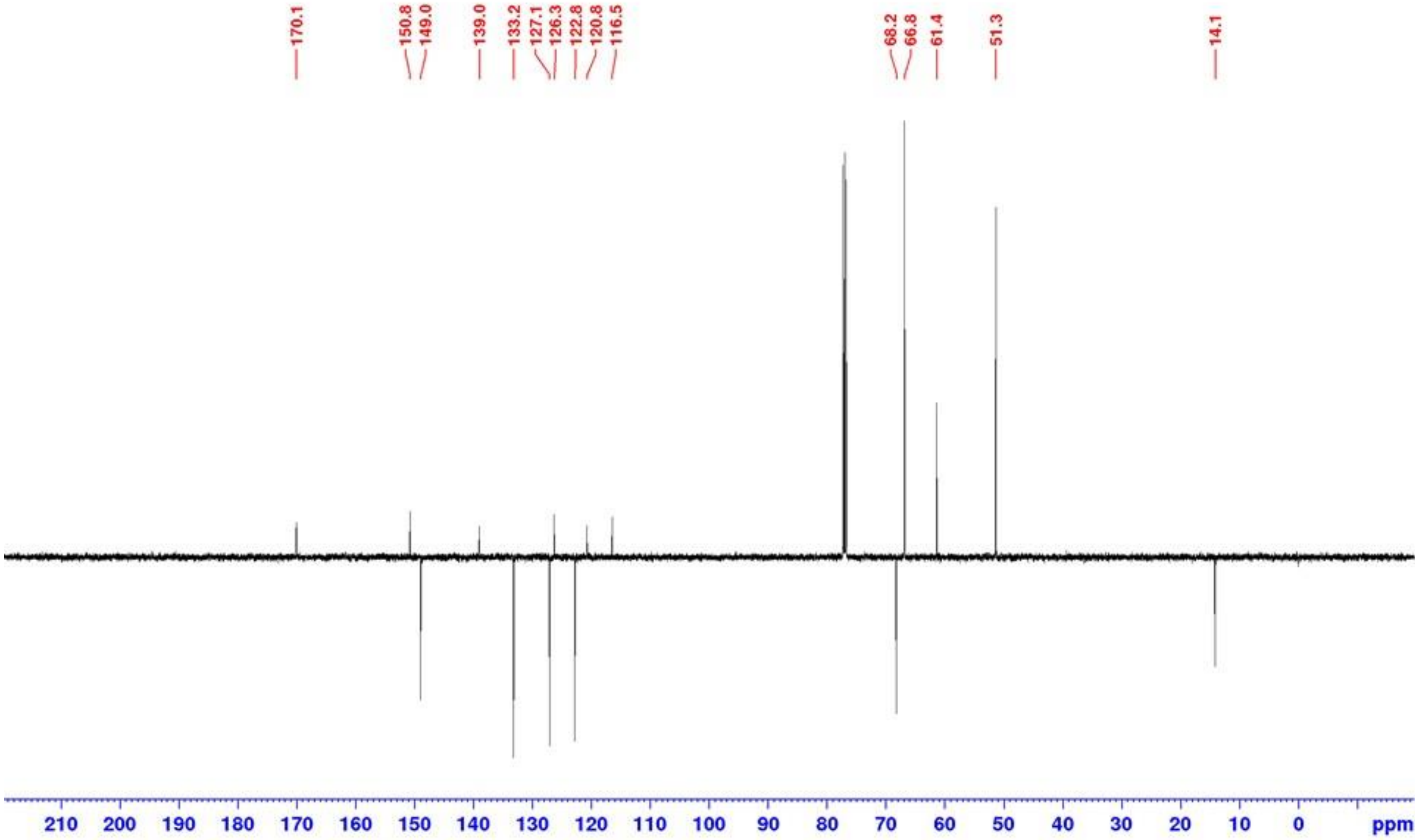

Figure S6. ^{13}C-NMR spectrum of **10**.

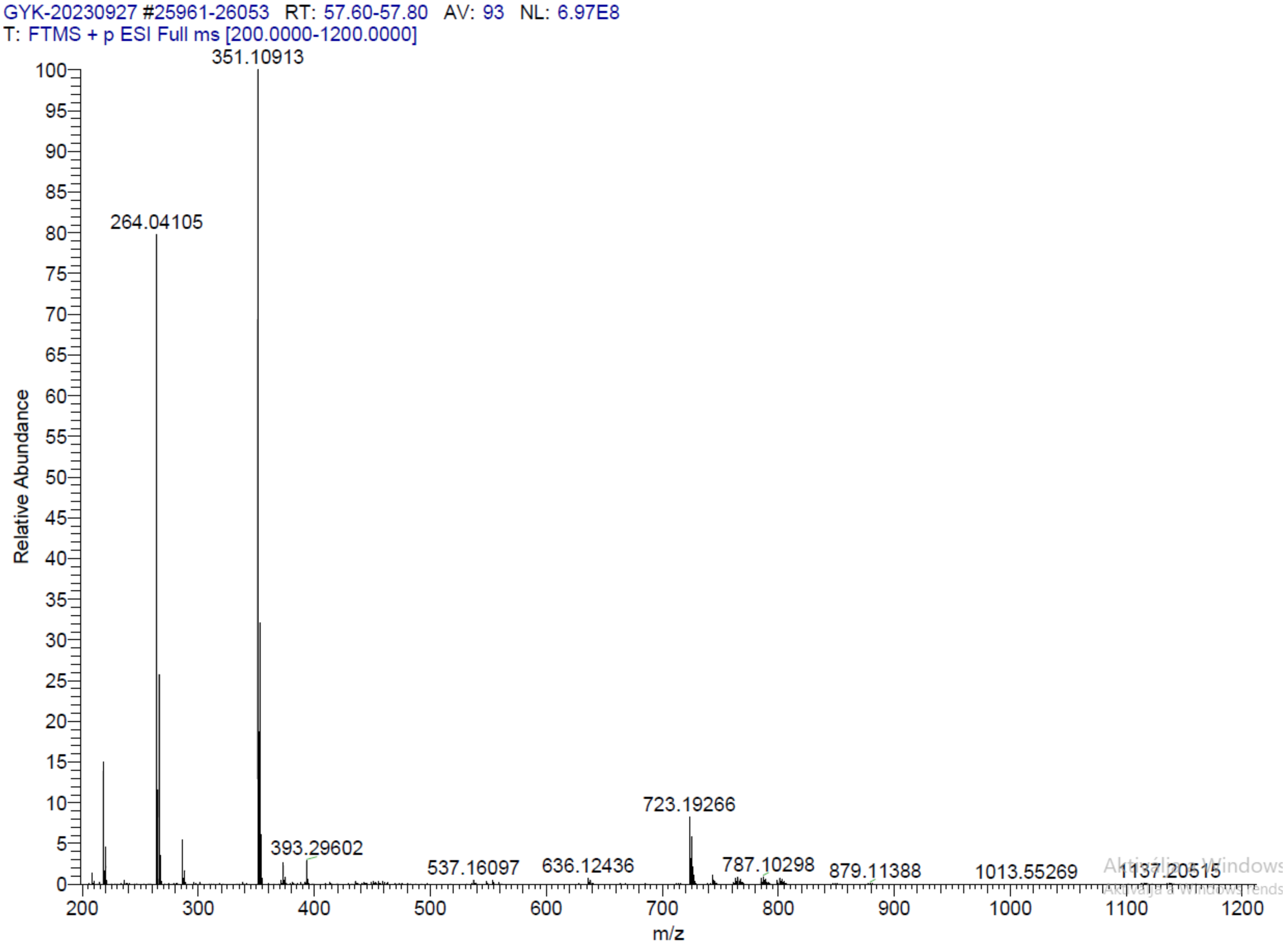

Figure S7. HRMS spectrum of **10** in positive mode.

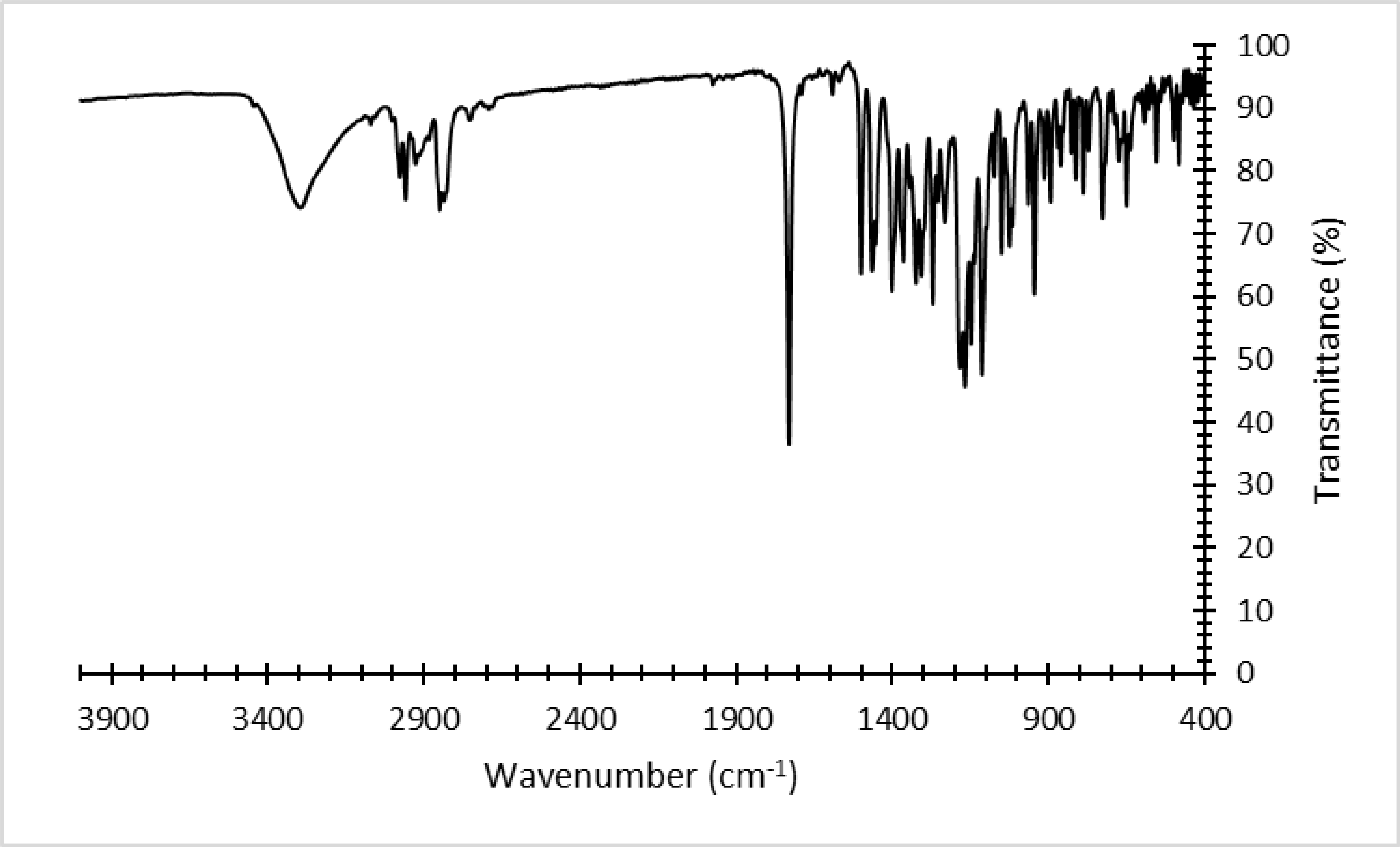

Figure S8. FTIR spectrum of **10**.

Melting point of **10**: 124-126 °C.

Ethyl 2-(5-chloro-8-hydroxyquinolin-7-yl)-2-(1*H*-indol-3-yl)acetate (11)

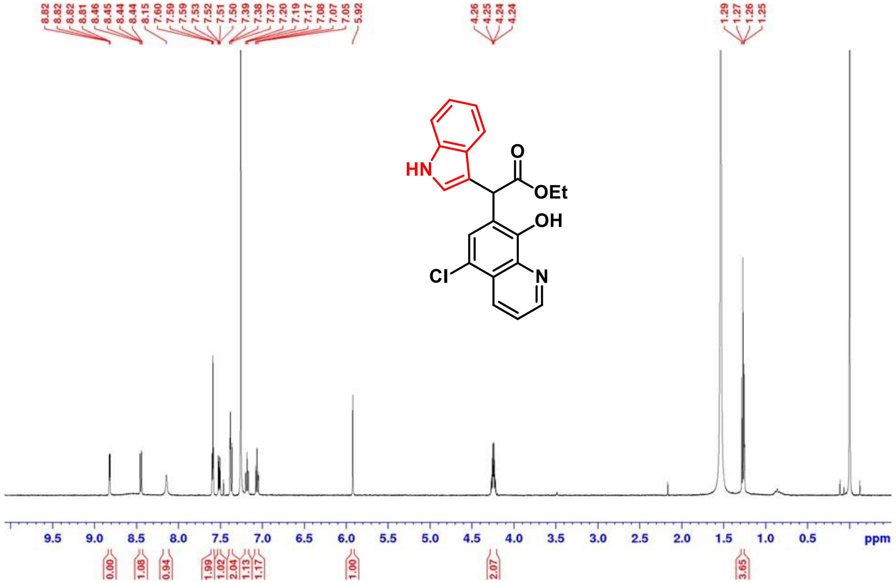

Figure S9. ^{1}H-NMR spectrum of **11**.

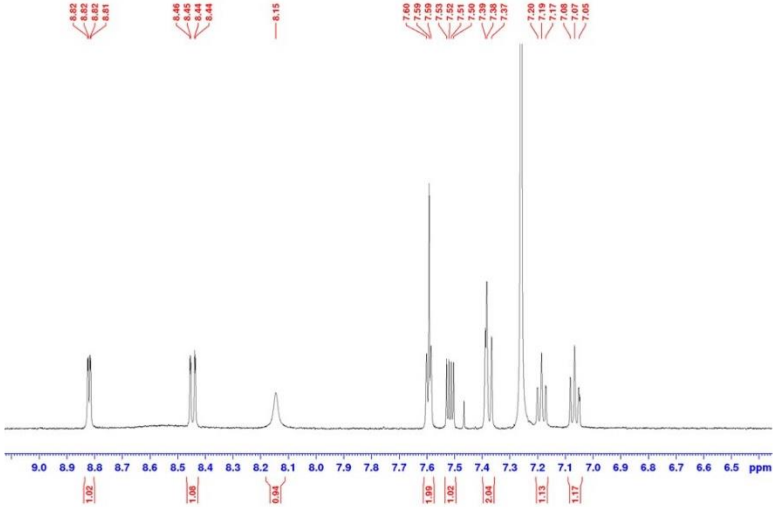

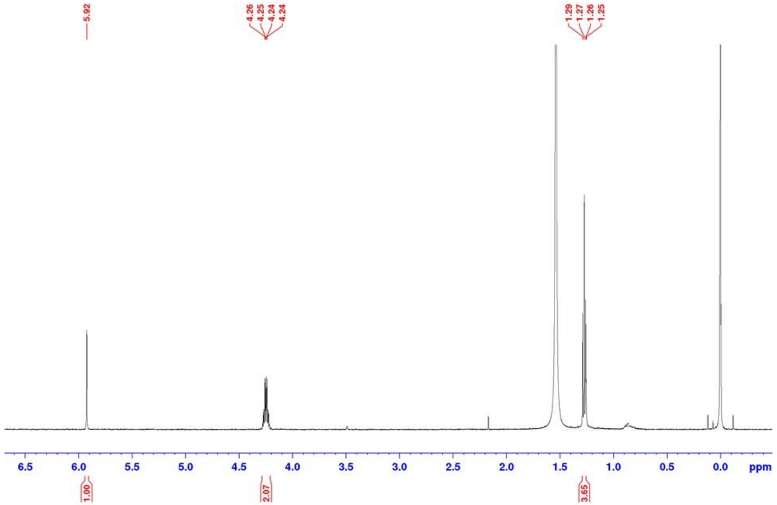

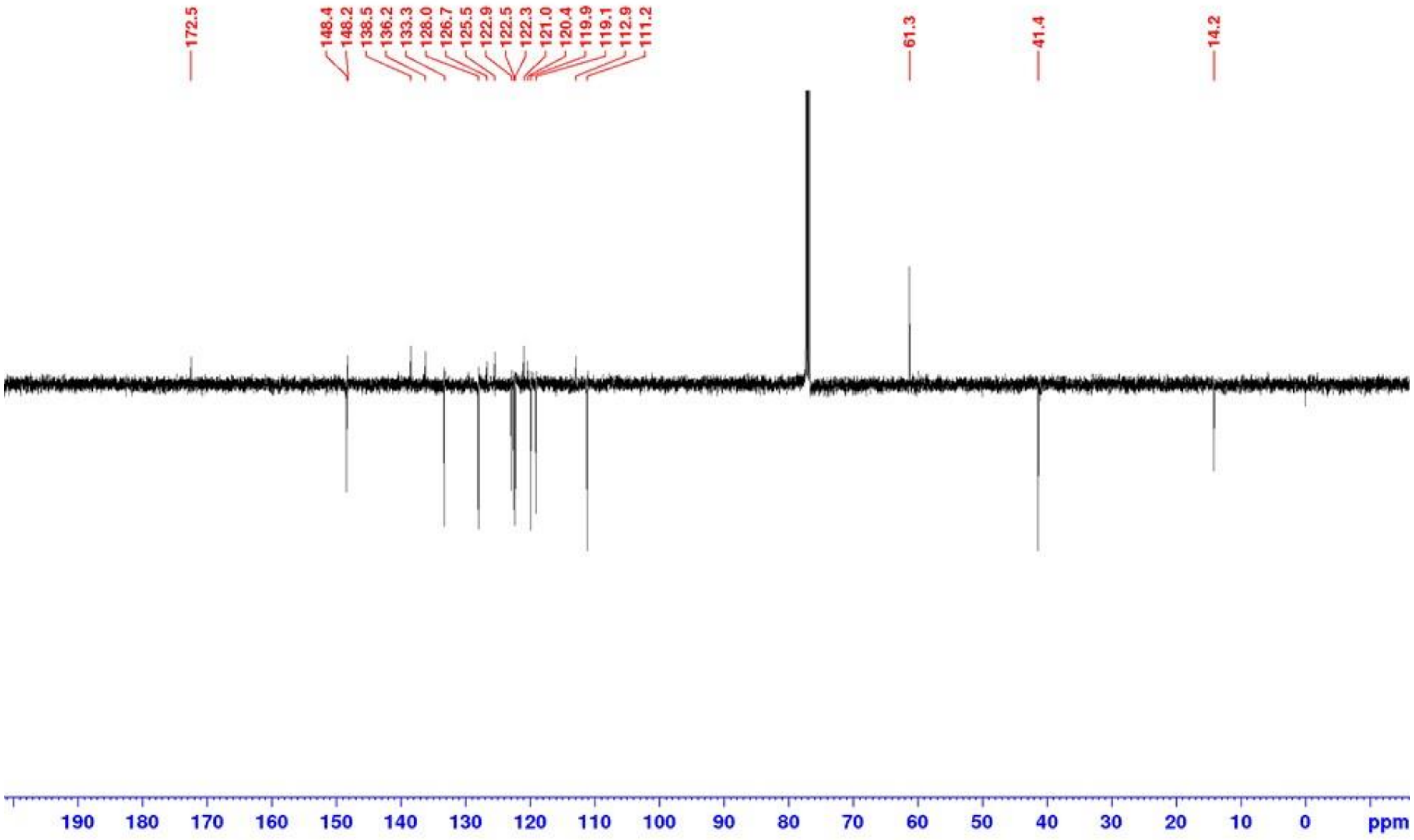

Figure S10. ^{13}C-NMR spectrum of **11**.

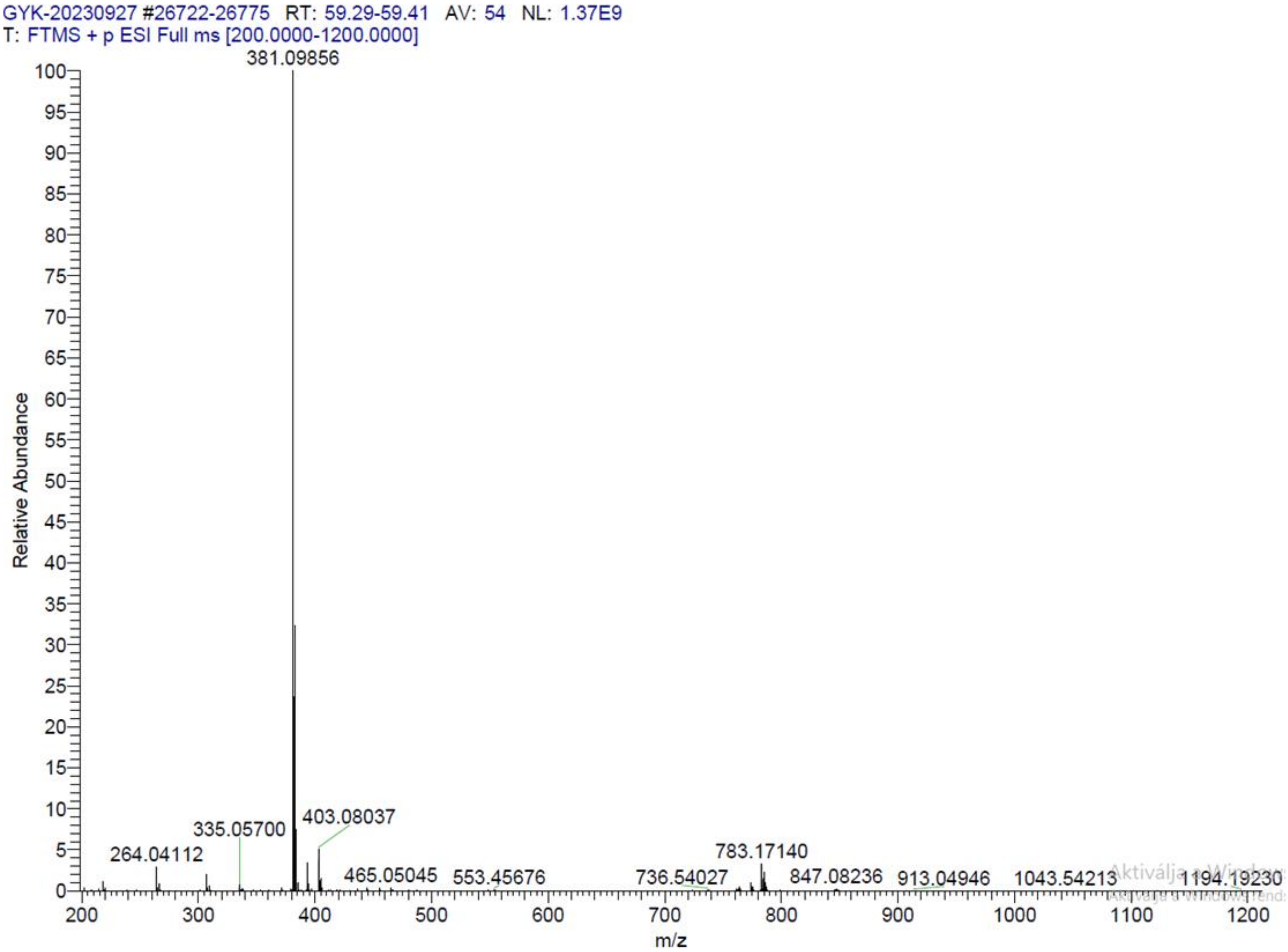

Figure S11. HRMS spectrum of **11** in positive mode.

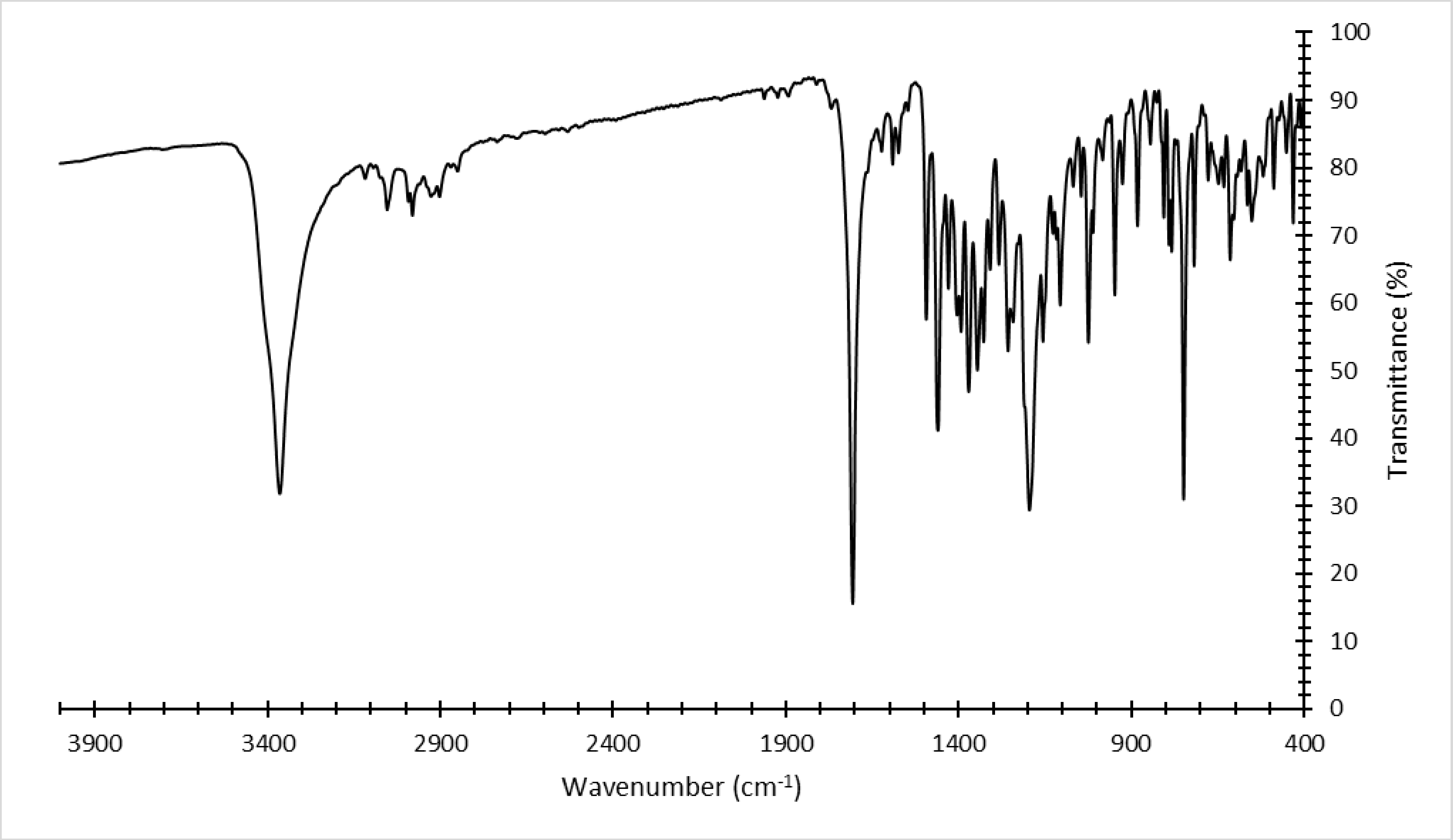

Figure S12. FTIR spectrum of **11**.

Ethyl 2-(5-chloro-8-hydroxyquinolin-7-yl)-2-(7-azaindole-3-yl)acetate (12)

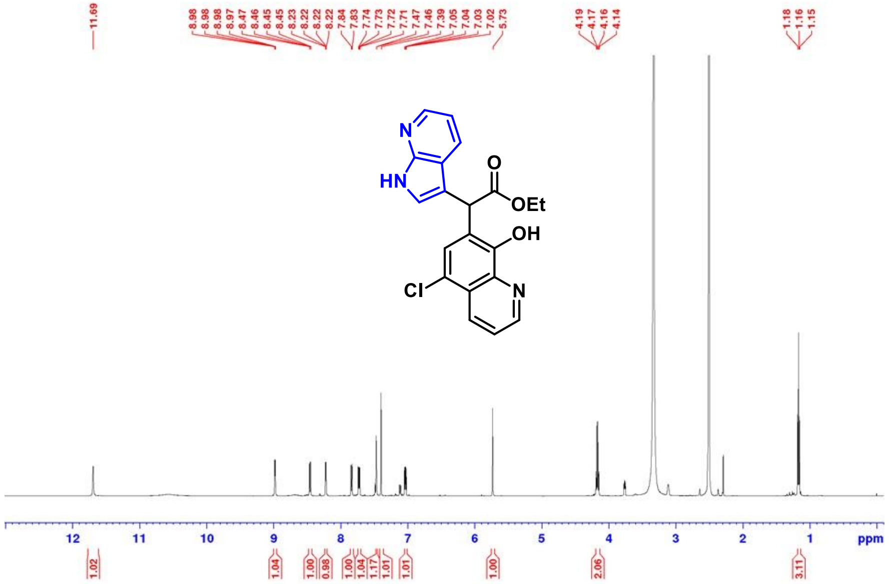

Figure S13. ^{1}H-NMR spectrum of **12**.

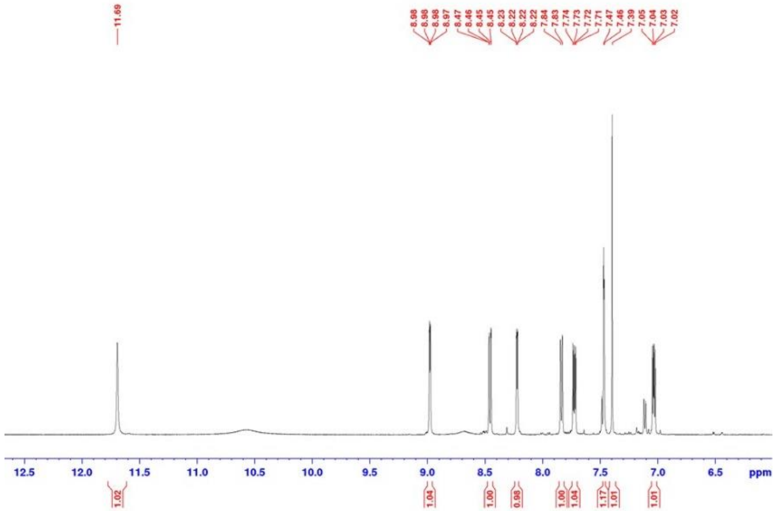

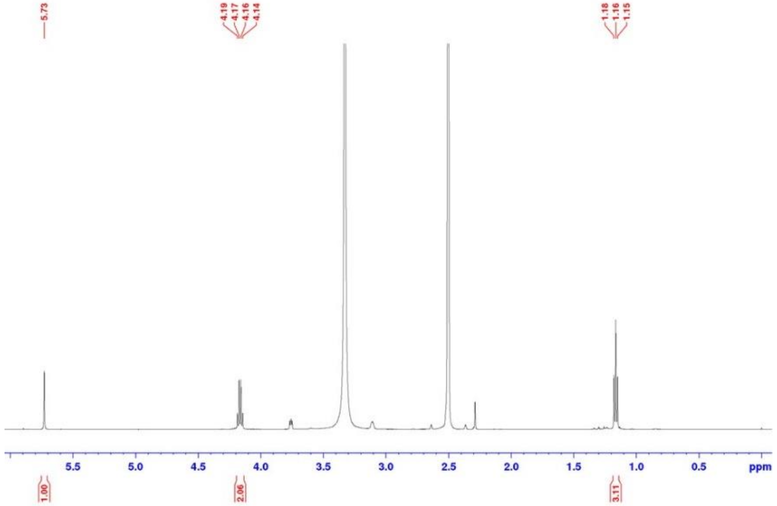

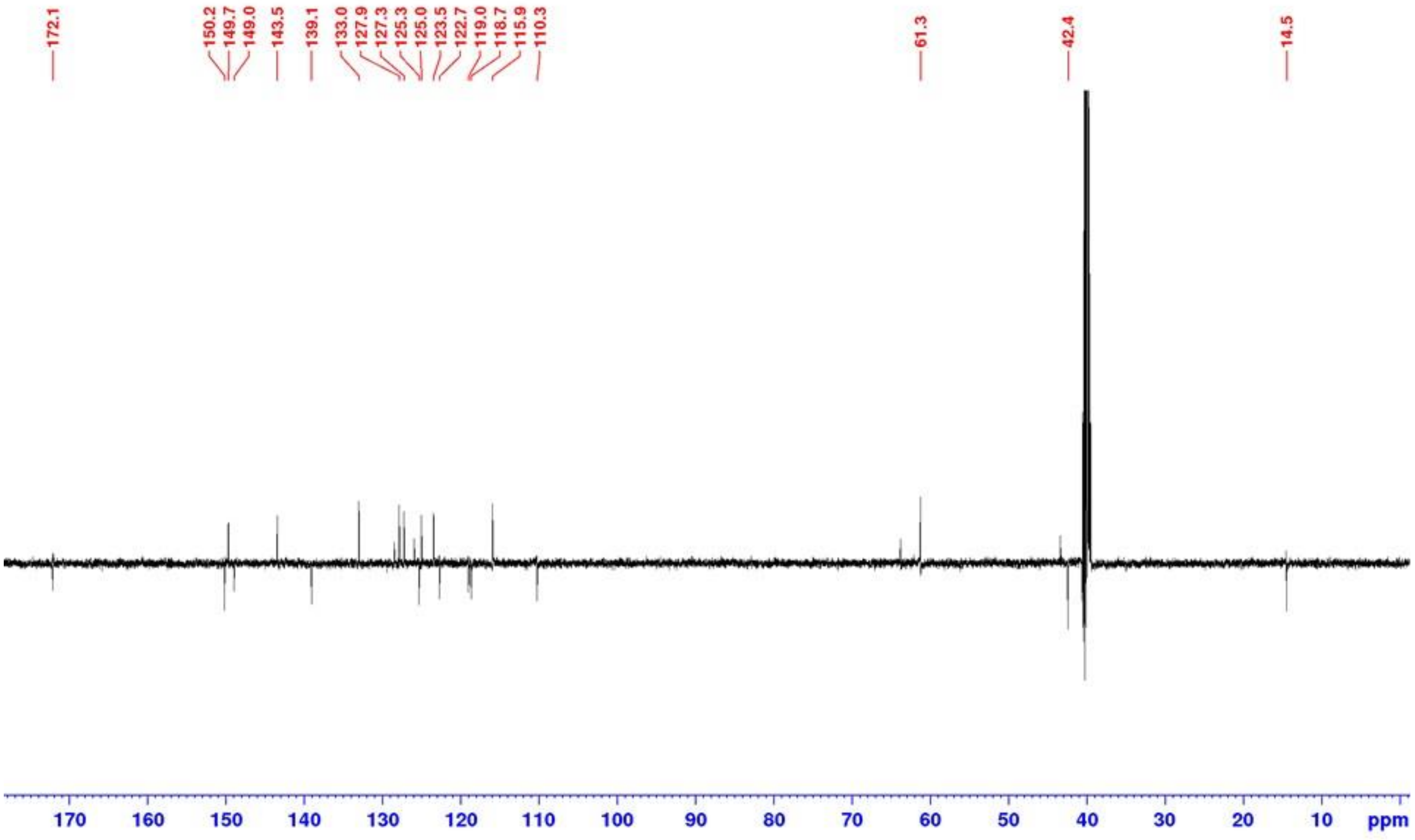

Figure S14. ¹³C-NMR spectrum of **12**.

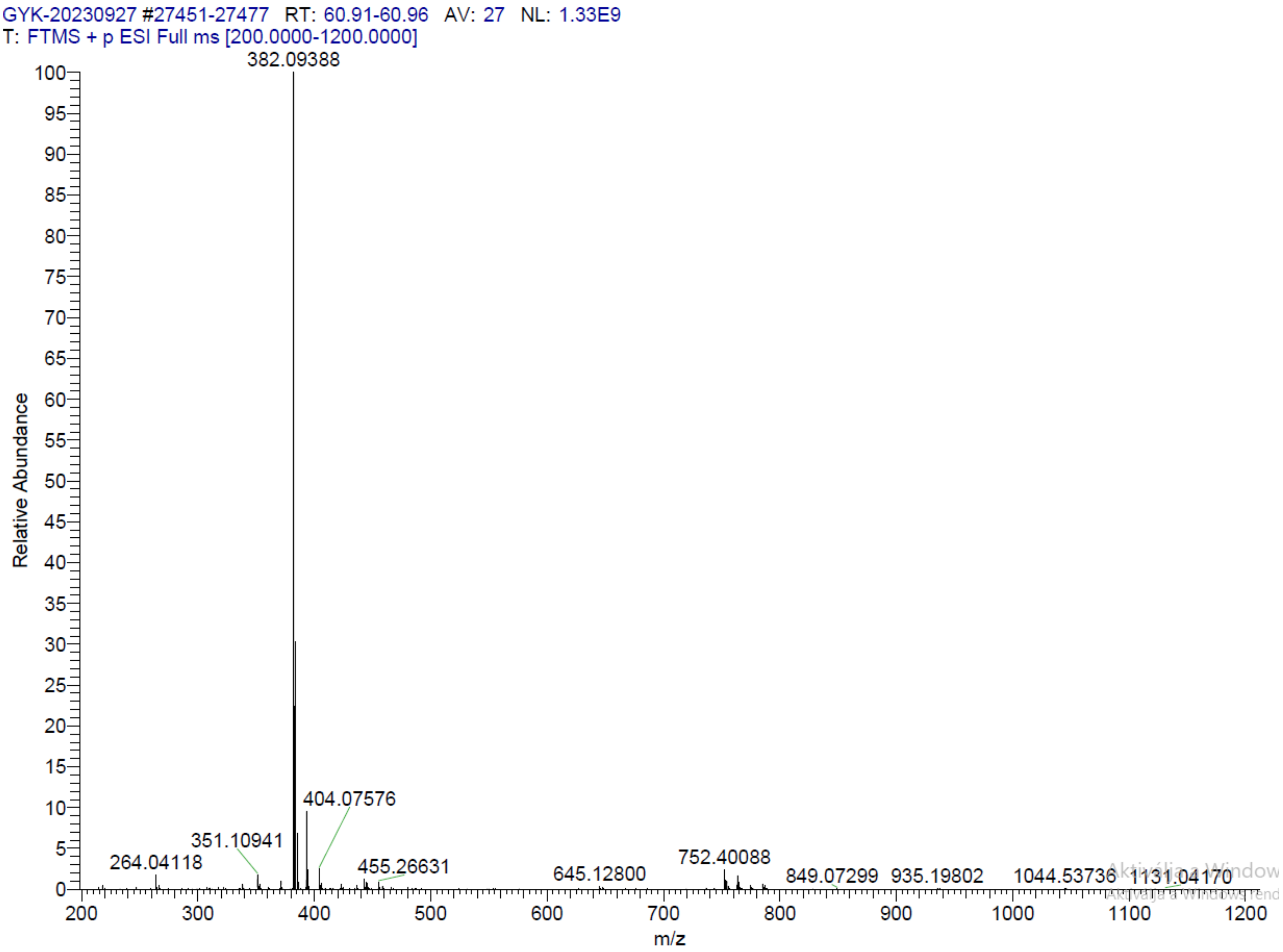

Figure S15. HRMS spectrum of **12** in positive mode.

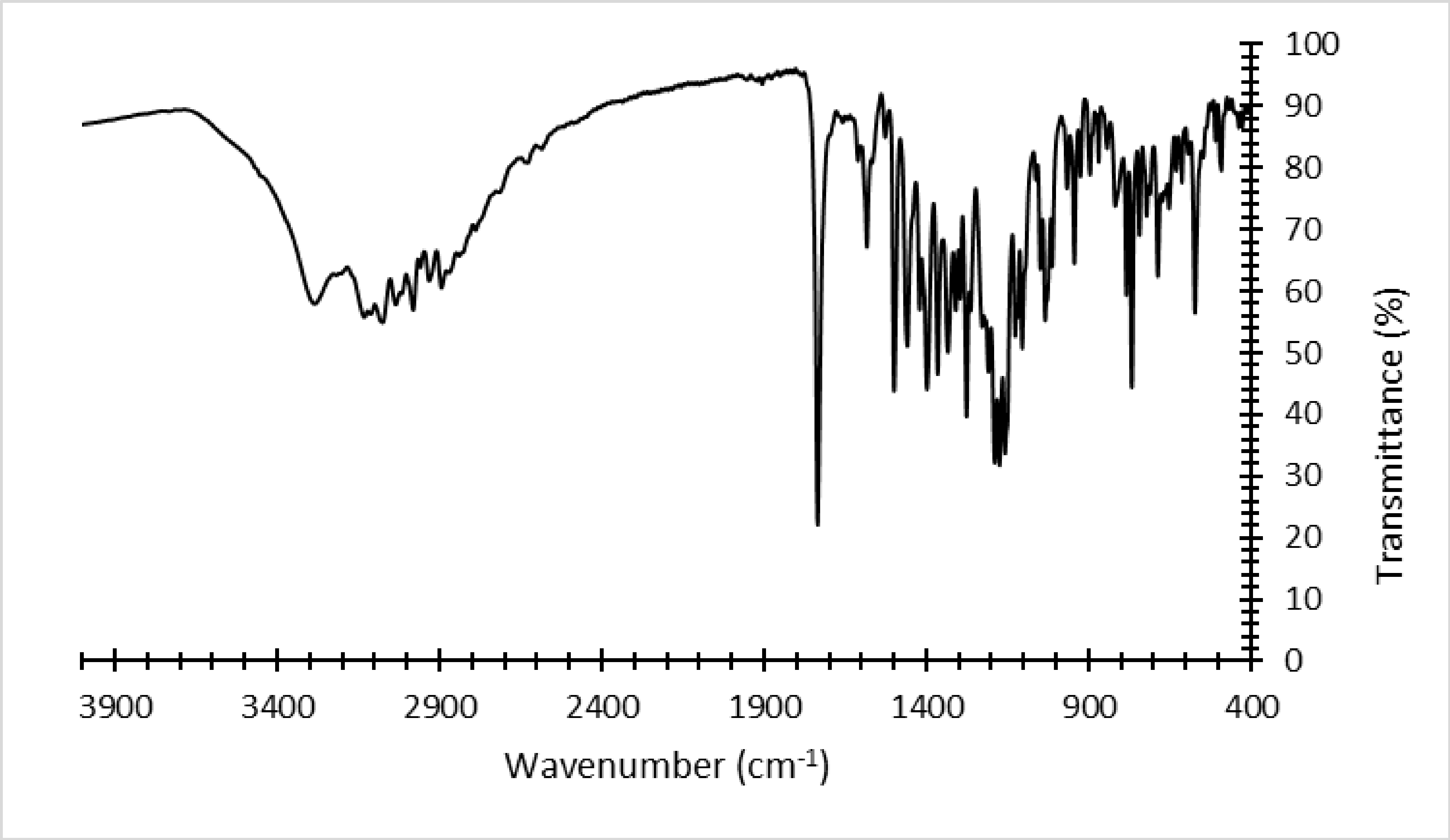

Figure S16. FTIR spectrum of **12**.

Melting point of **12**: 228-230 °C

7-((1*H*-indol-3-yl)(phenyl)methyl)-5-chloroquinolin-8-ol (14)

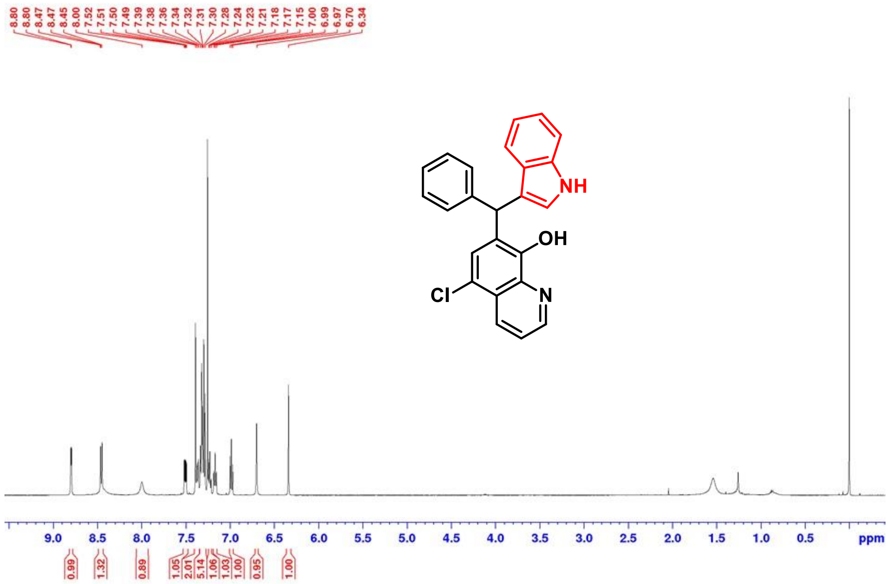

Figure S17. ¹H-NMR spectrum of **14**.

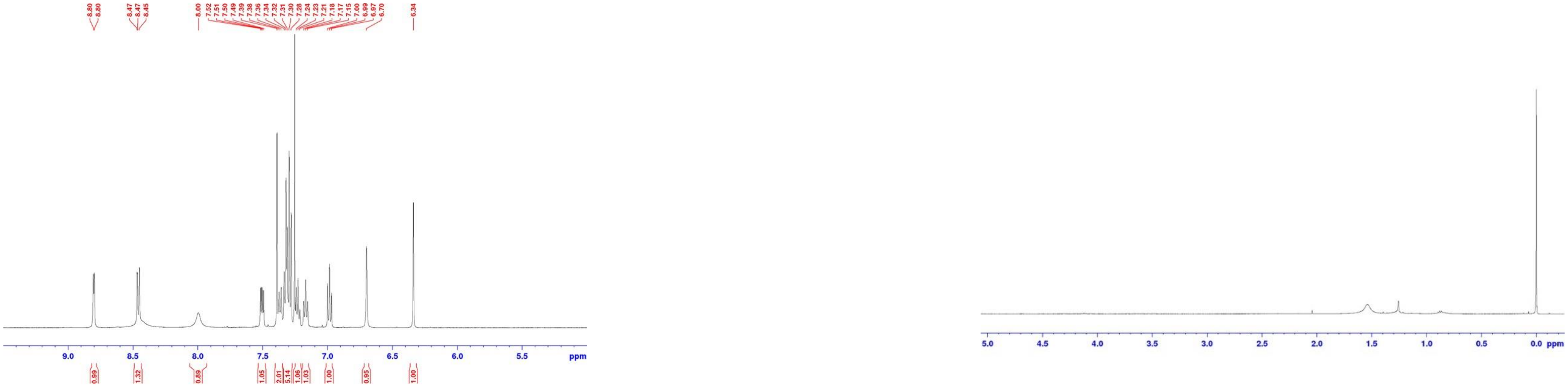

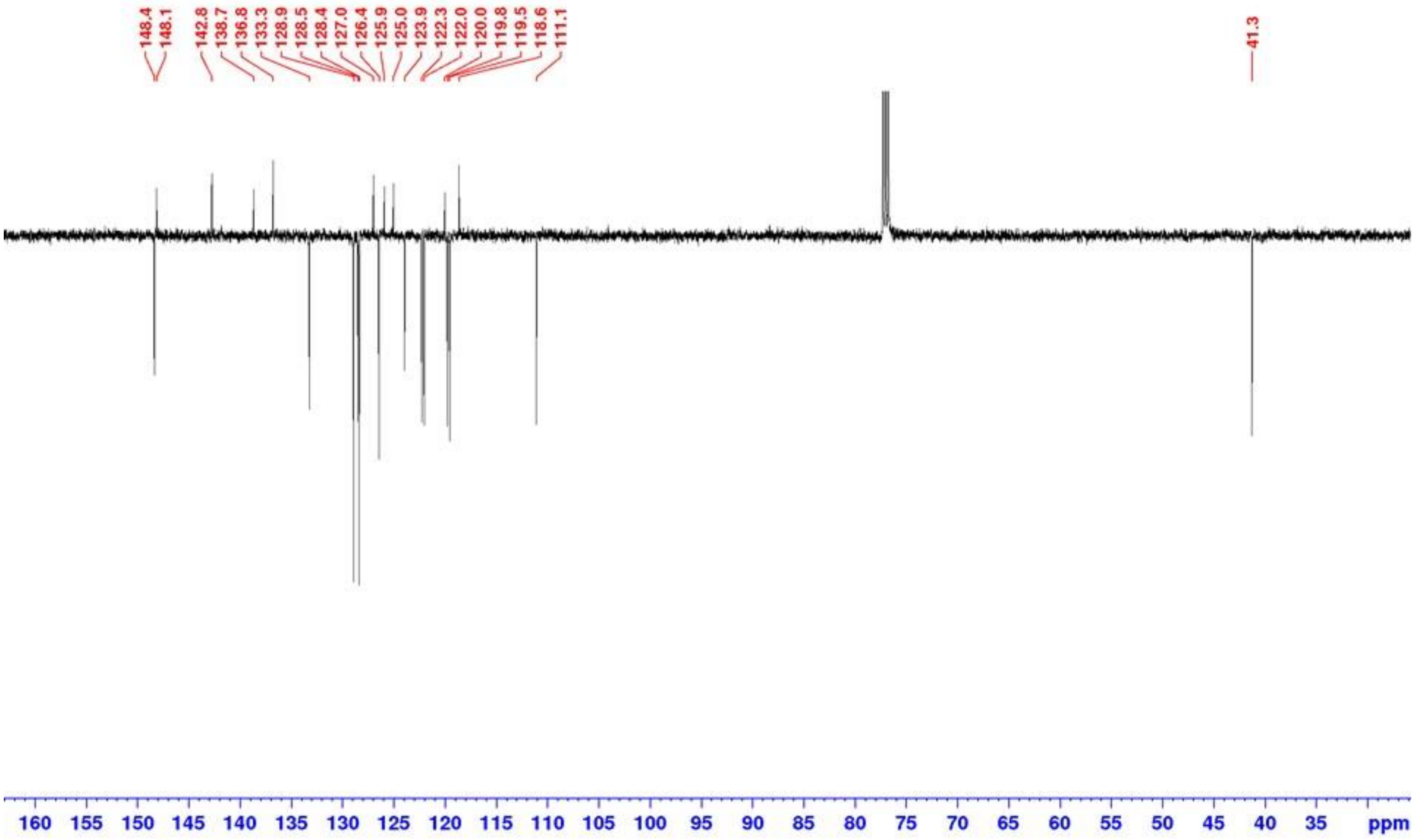

Figure S18. ^{13}C-NMR spectrum of **14**.

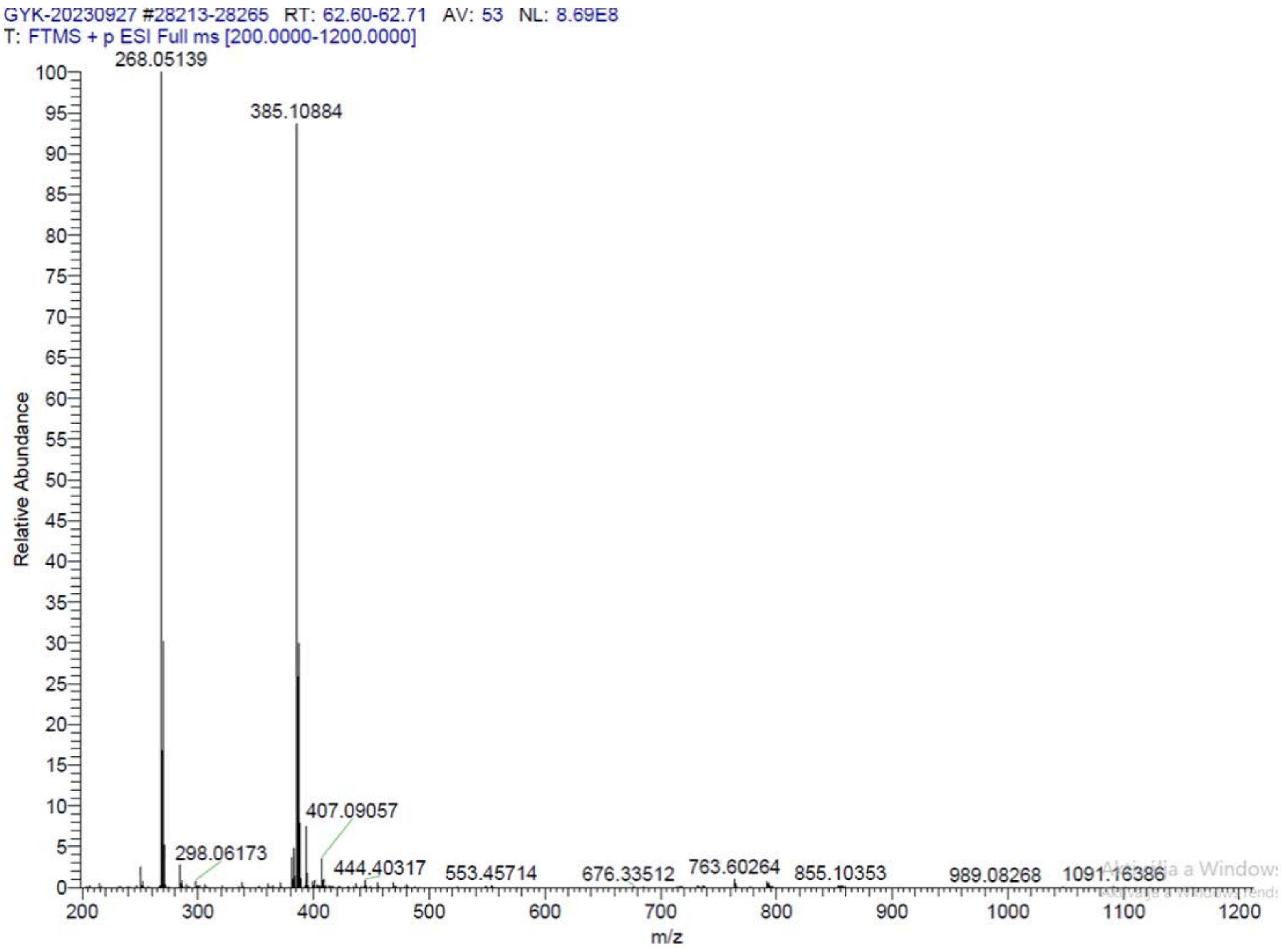

Figure S19. HRMS spectrum of **14** in positive mode.

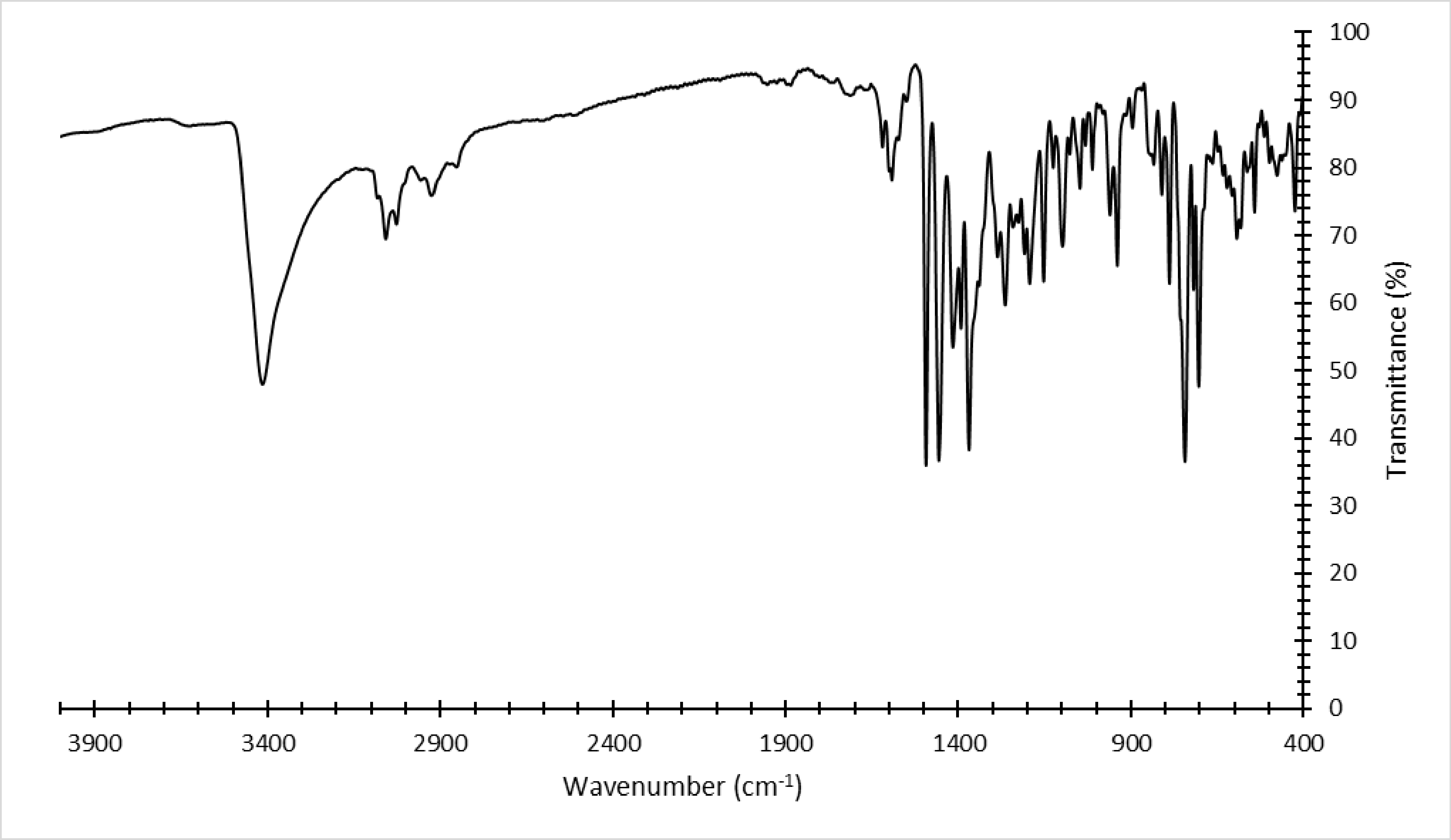

Figure S20. FTIR spectrum of **14**.

7-((1*H*-indol-3-yl)methyl)-5-chloroquinolin-8-ol (16)

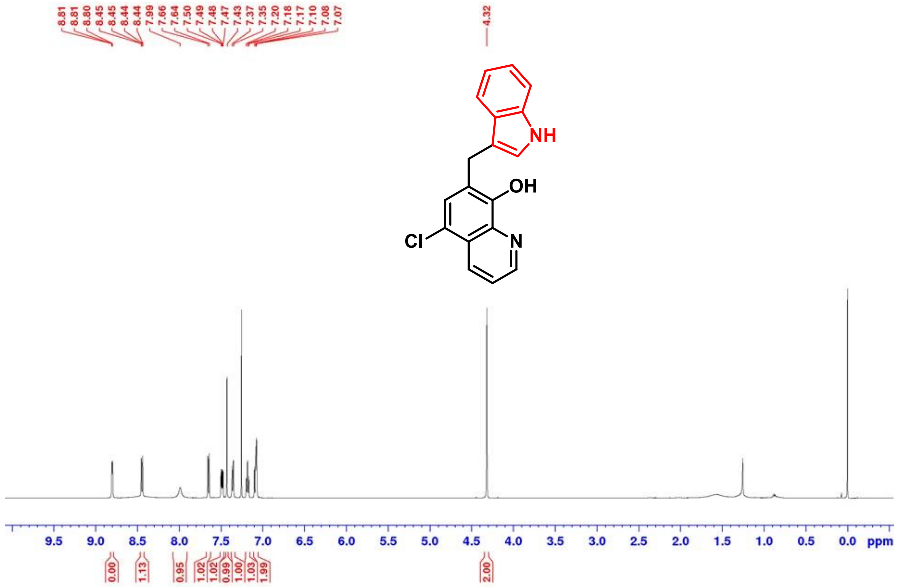

Figure S21. ^{1}H-NMR spectrum of **16**.

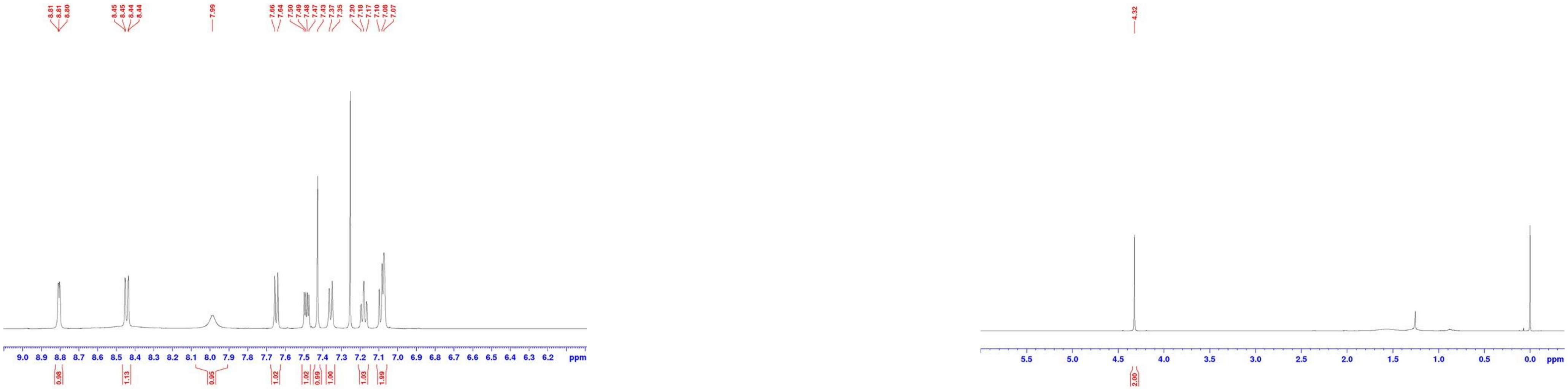

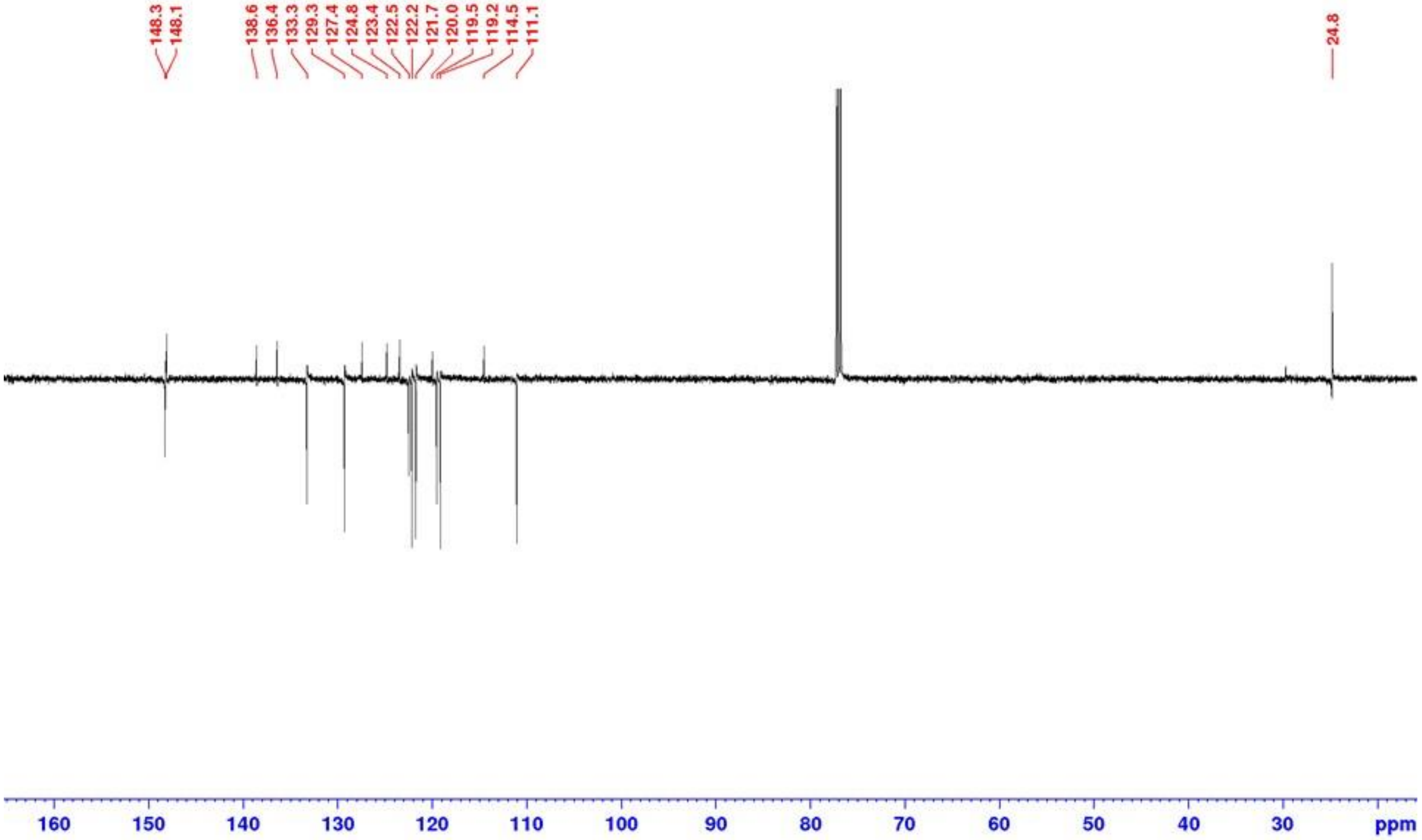

Figure S22. ^{13}C-NMR spectrum of **16**.

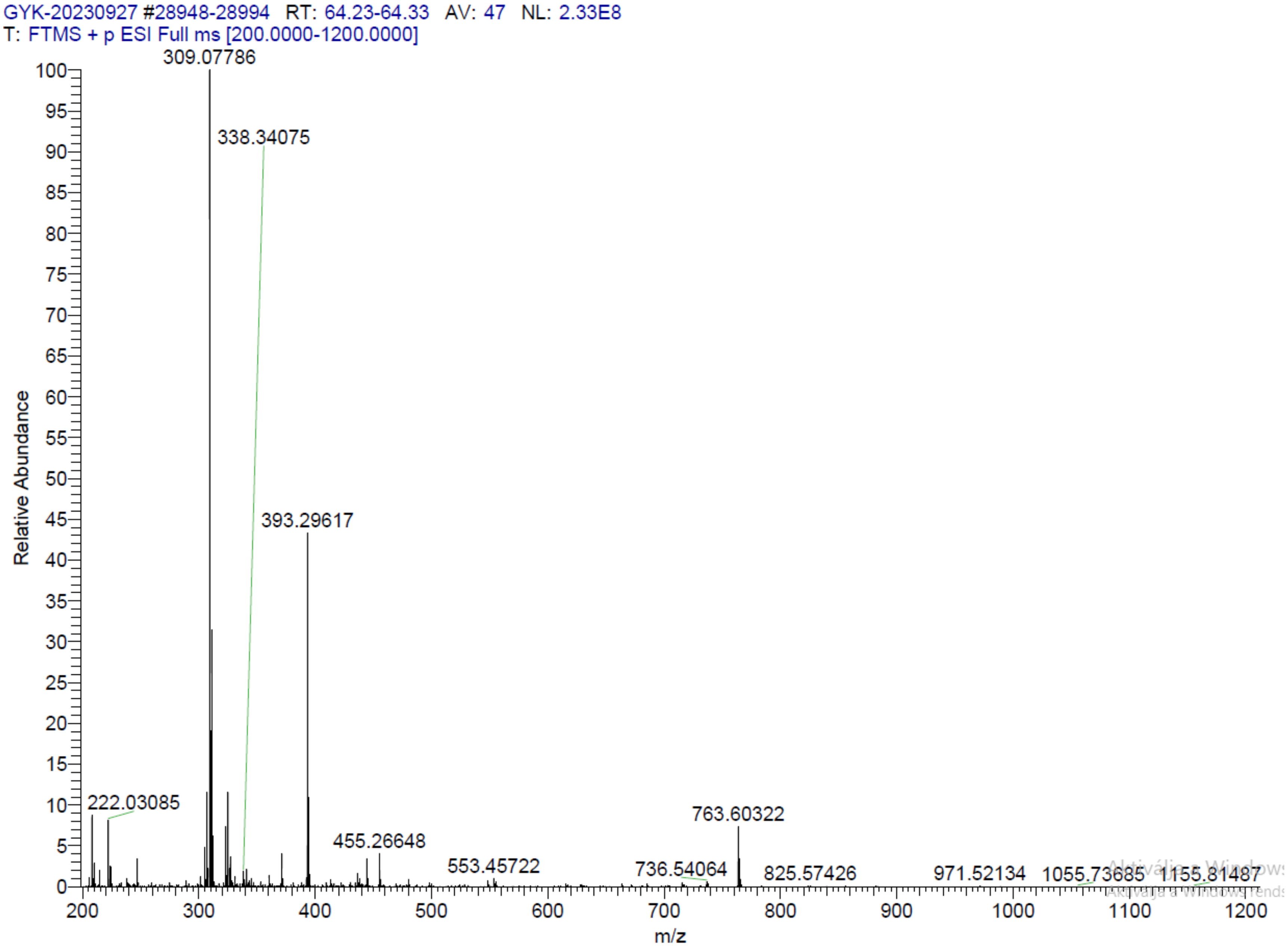

Figure S23. HRMS spectrum of **16** in positive mode.

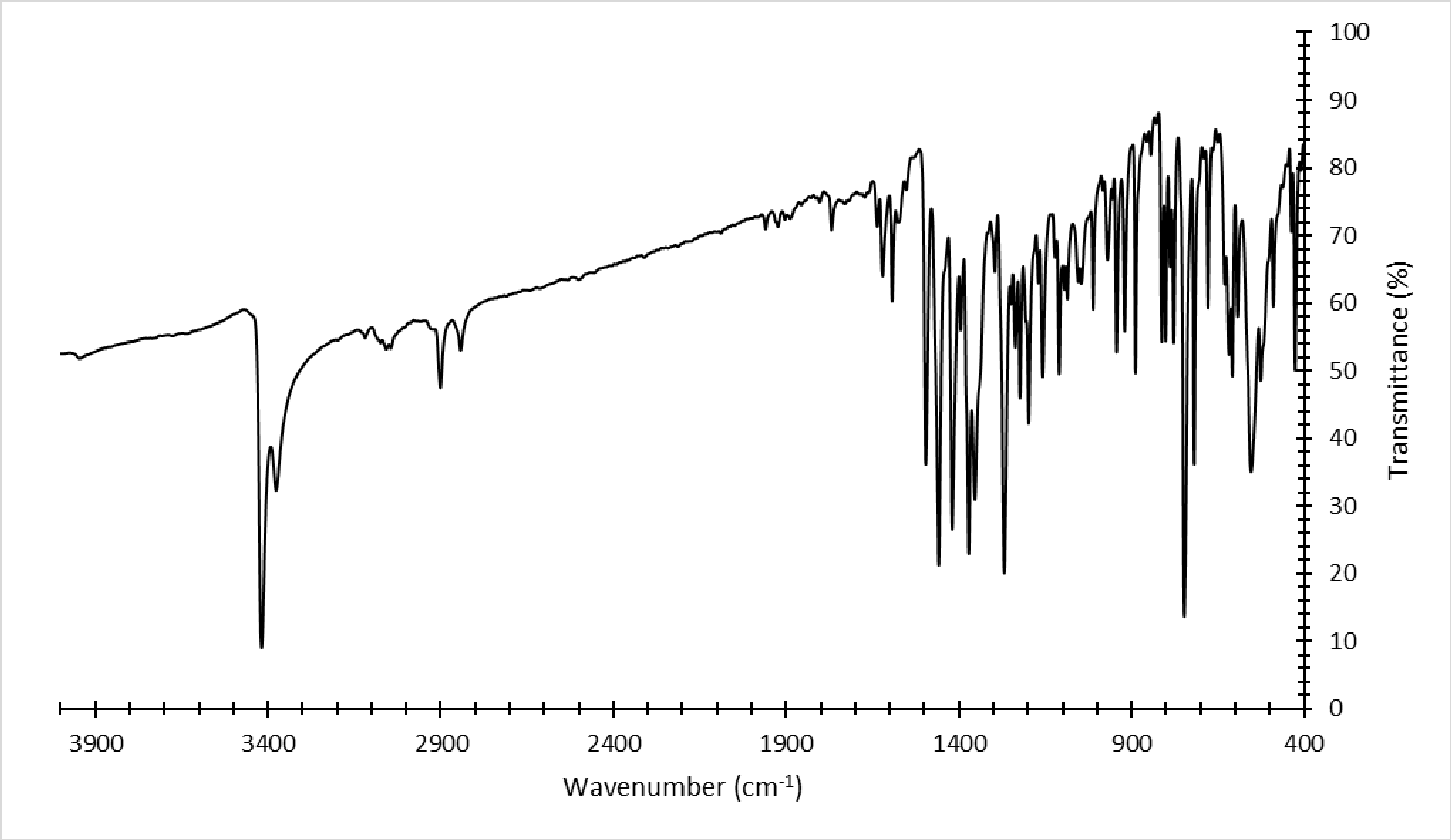

Figure S24. FTIR spectrum of **16**.

7-((7-azaindole-3-yl)methyl)-5-chloroquinolin-8-ol (17)

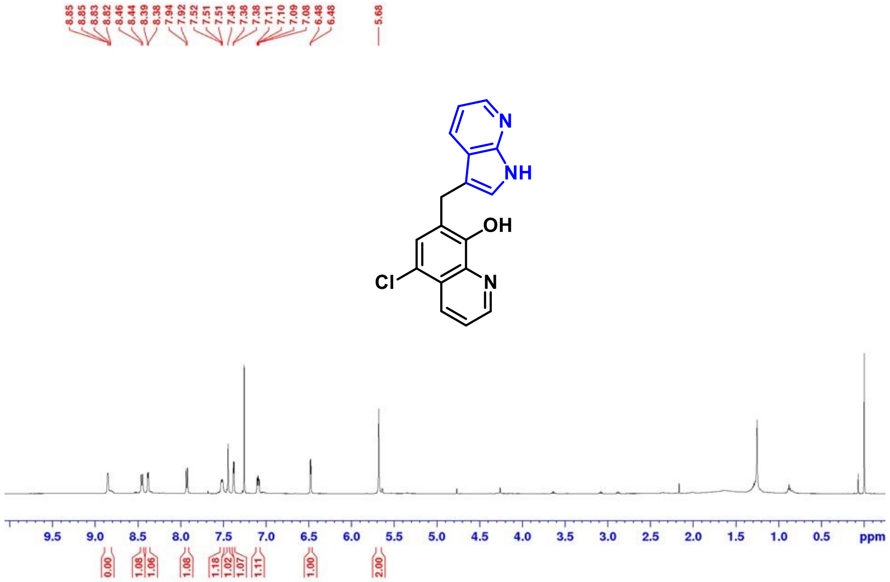

Figure S25. ^{1}H-NMR spectrum of **17**.

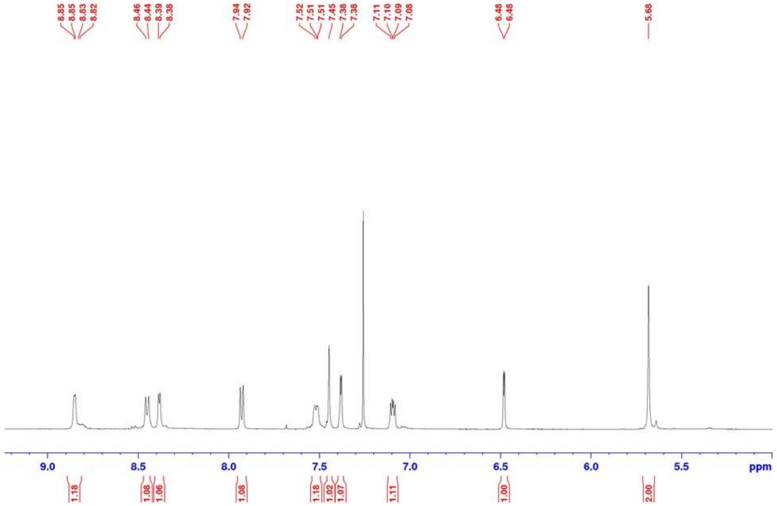

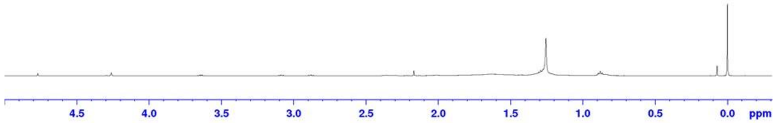

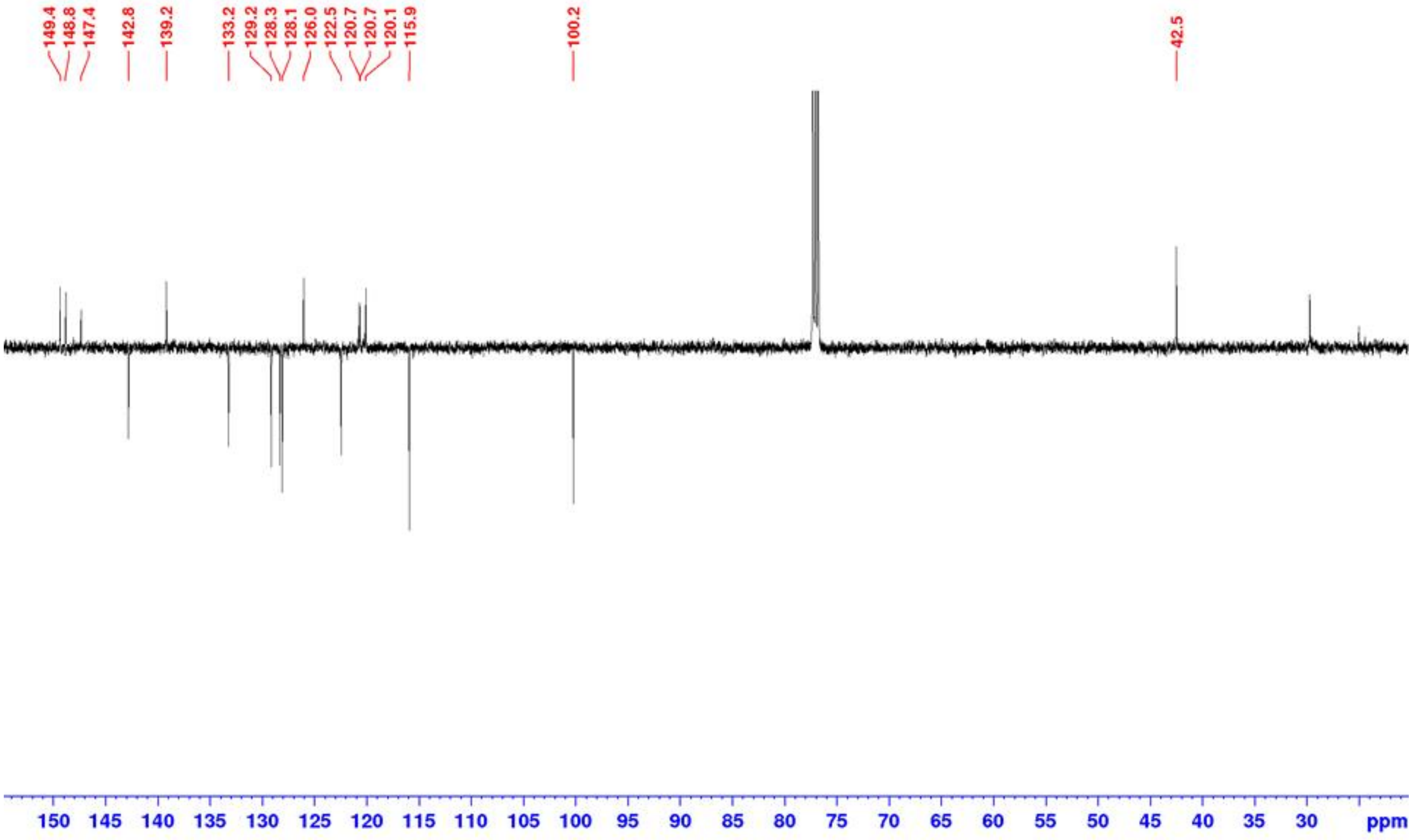

Figure S26. ^{13}C-NMR spectrum of **17**.

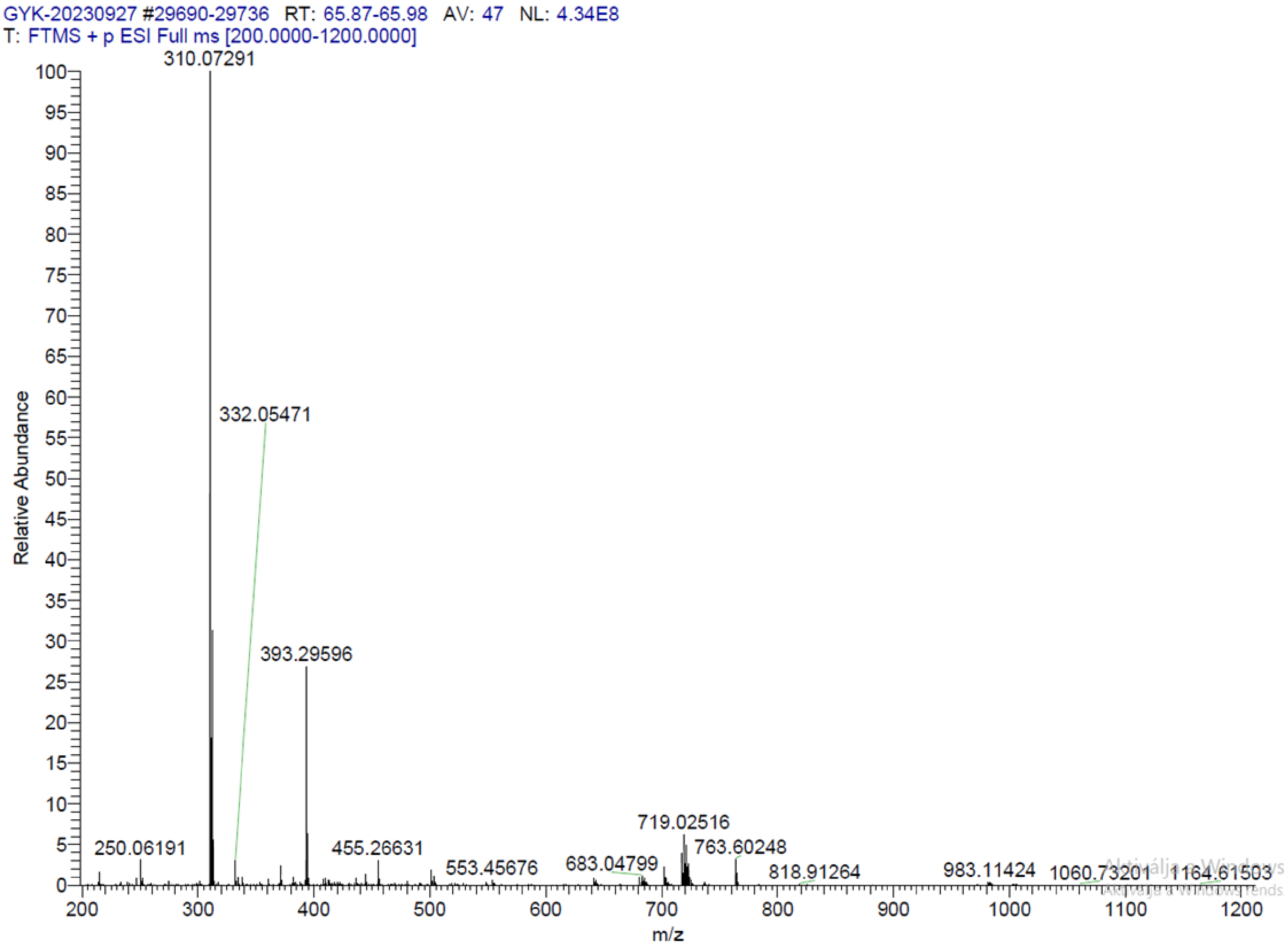

Figure S27. HRMS spectrum of **17** in positive mode.

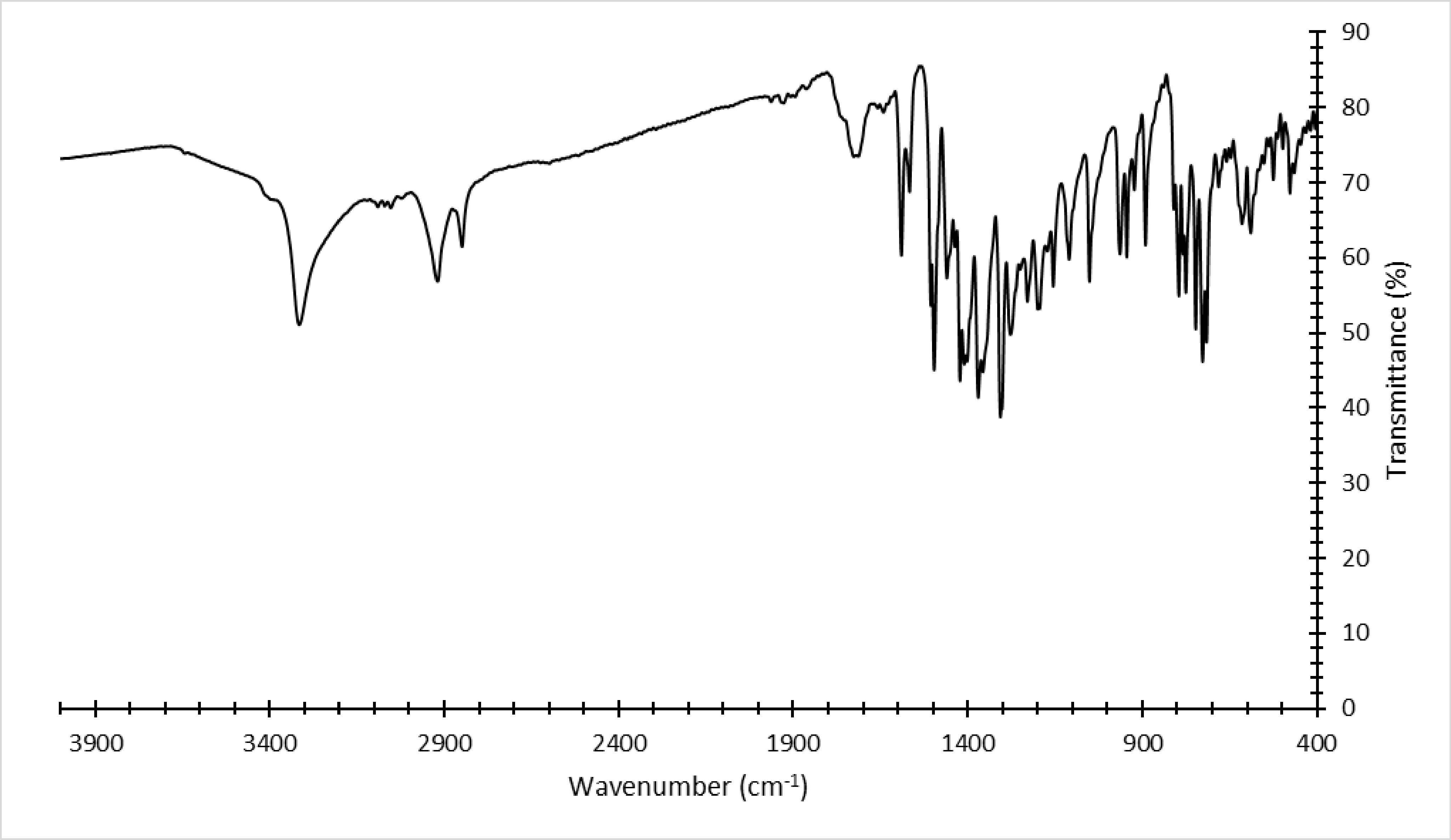

Figure S28. FTIR spectrum of **17**.
